# Supplementary figures and images for: The Discovery, Distribution, and Evolution of Viruses Associated with Drosophila melanogaster
Source: PLoS Biol. 2015 Jul 14;13(7):e1002210. doi: 10.1371/journal.pbio.1002210 (PMC4501690; doi:10.1371/journal.pbio.1002210)

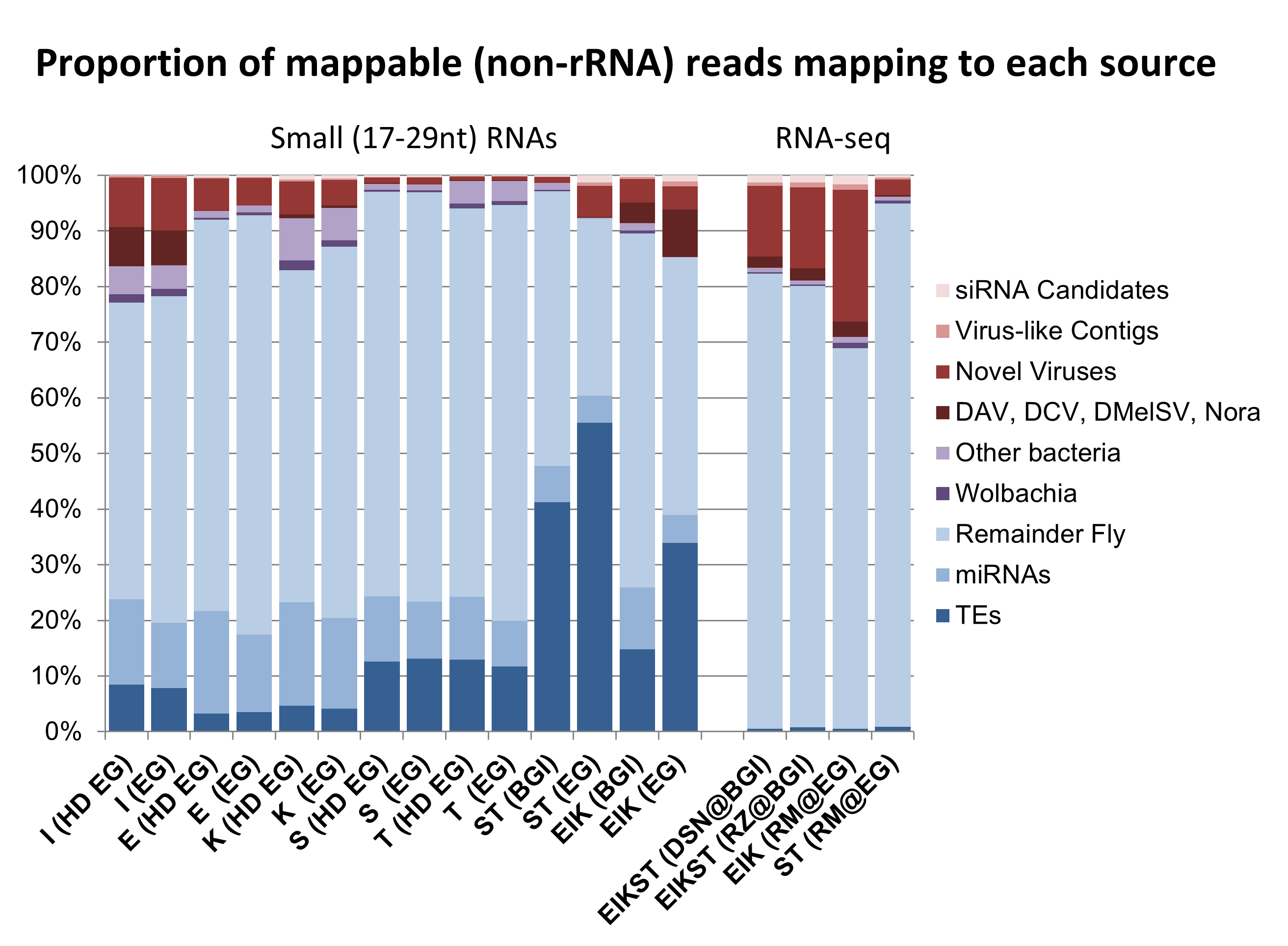

Supplement: S1 Fig — The proportion of RNAseq and small (17-29nt) reads that map to characterised D. melanogaster transposable elements, Drosophila miRNAs, the remainder of the D. melanogaster genome, Wolbachia and other bacteria, the four “classical” viruses (DAV, DCV, DMelSV, and Nora Virus), the new named viruses, other BLAST-candidate viruses, and siRNA-candidate viruses. Counts exclude unmapped reads, and reads mapping to Drosophila ribosomal sequences. EIKST refers to the metagenomic pools or mixtures thereof, “BGI” and “EG” indicate sequencing was performed by the Beijing Genomics Institute or Edinburgh Genomics (respectively), “HD” indicates the use of “High Definition” ligation adapters for small RNA sequencing, “RM” and “RZ” indicate the use of rRNA depletion by RiboMinus or Ribo-Zero, and “DSN” indicates double-stranded nuclease normalisation. Raw counts data are provided in S1 Table. (TIF) [file pbio.1002210.s010.tif]

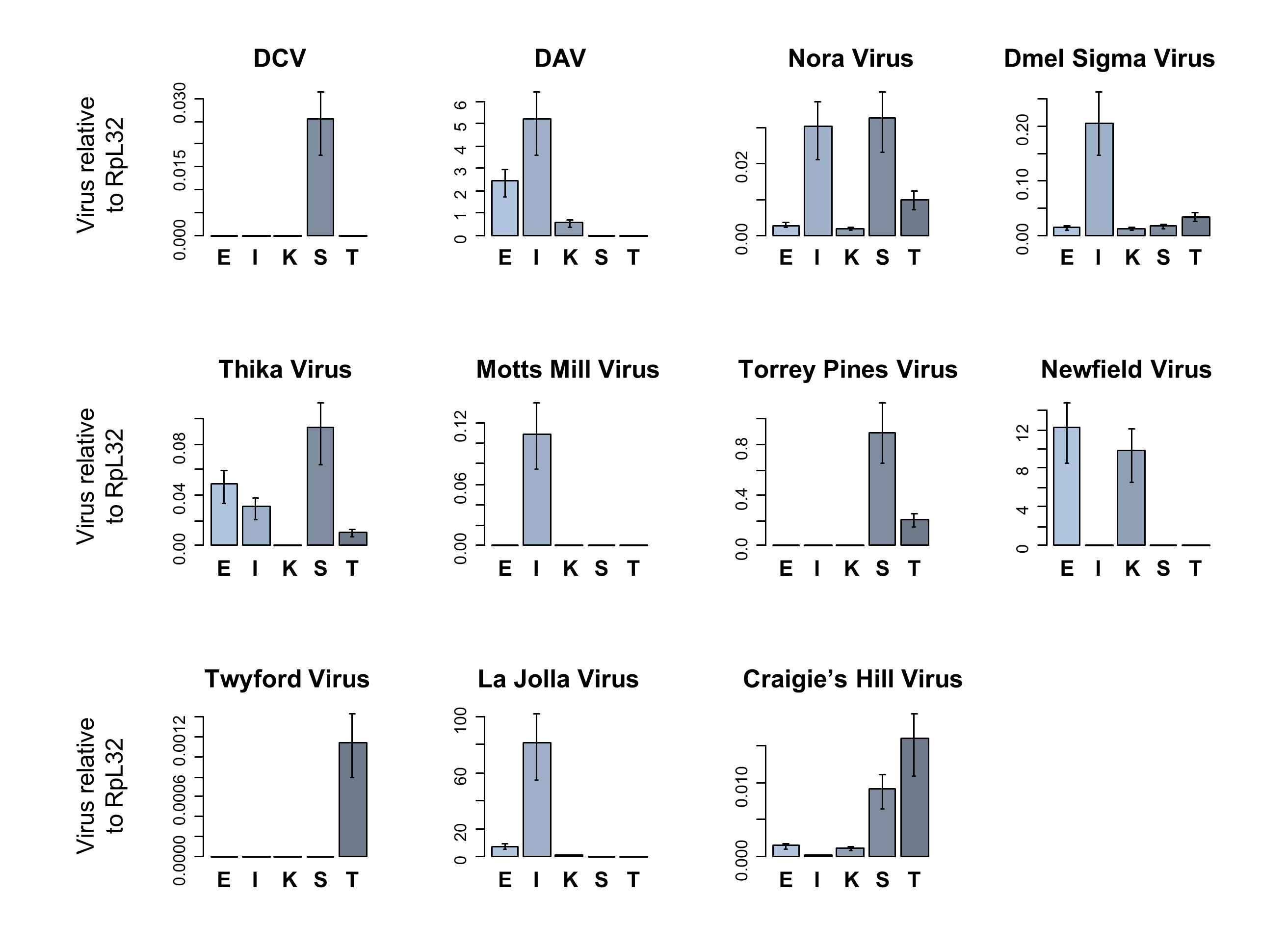

Supplement: S2 Fig — RNA quantification for DCV, DAV, Nora Virus, DMelSV, Thika Virus, Motts Mill Virus, Torrey Pines Virus, Newfield Virus, Twyford Virus, La Jolla Virus and Craigie’s Hill Virus present in each of the five metagenomic pools E, I, K, S, and T, quantified by qRT-PCR relative to the Drosophila ribosomal protein gene RpL32. Note that DCV and Twyford Virus were each detectable in a single pool. Error bars represent 80% credibility intervals for means, based on two or three replicates per sample and assuming the efficiencies inferred from dilution series (S2 Text). Virus presence/absence agrees well with small RNA data (S1 Fig) but qRT-PCR quantification may be unreliable given the high sequence diversity in these virus pools. Raw CT data are given in S1 Data. (TIF) [file pbio.1002210.s011.tif]

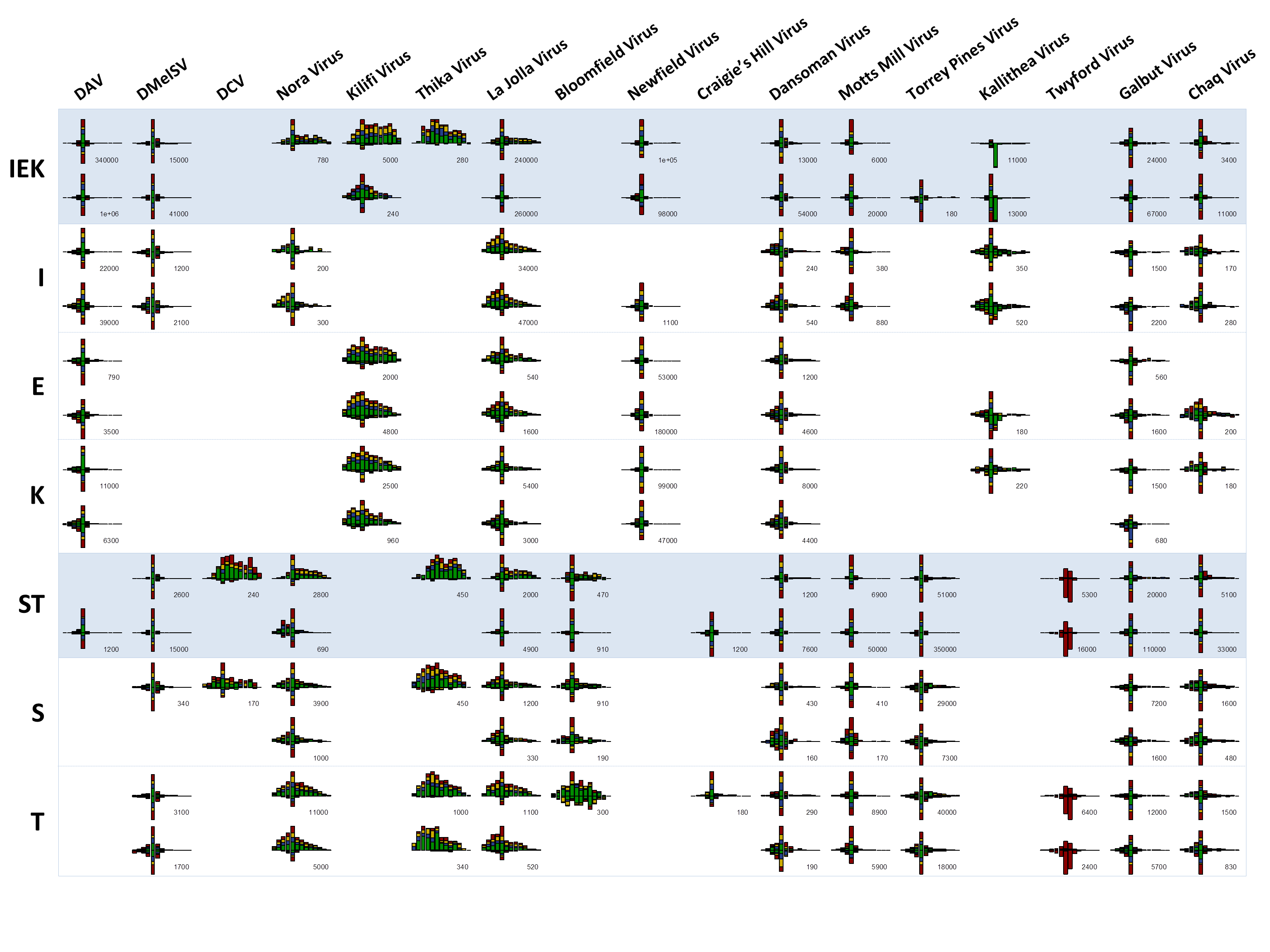

Supplement: S4 Fig — The bar plots show the size distribution of small RNAs (17–29 nt) for selected viruses (columns) separated across the 14 different sequencing libraries (rows). Bars plotted above the x-axis represent reads mapping to the positive strand and those below represent reads mapping to the negative strand. Bars are coloured according to the proportion of reads with each 5′-base (A-green, C-blue, G-yellow, U-red). The approximate number of reads for each virus in each pool is shown inset, and only viruses that had >100 small RNA reads in that pool are shown. Pairs of rows show technical replicates, and are labelled by metagenomic pool (E, I, K, S, T) or pool-mixture. For mixed pools (blue background) the reads in the lower rows were sequenced by Edinburgh Genomics following an oxidation step (to reduce miRNA representation), and the upper rows by BGI without an oxidation step. For unmixed pools (white background) the reads in the lower rows were sequenced using a “High Definition” ligation protocol (both without oxidation). Note that the relative number of longer (23–27 nt) reads seen in DCV, Nora Virus, Kilifi Virus, and Thika Virus appears to be reduced in the presence of an oxidation step, as is the number of Kallithea miRNA reads. Raw count data for this figure are provided in S1 Data. (TIF) [file pbio.1002210.s013.tif]

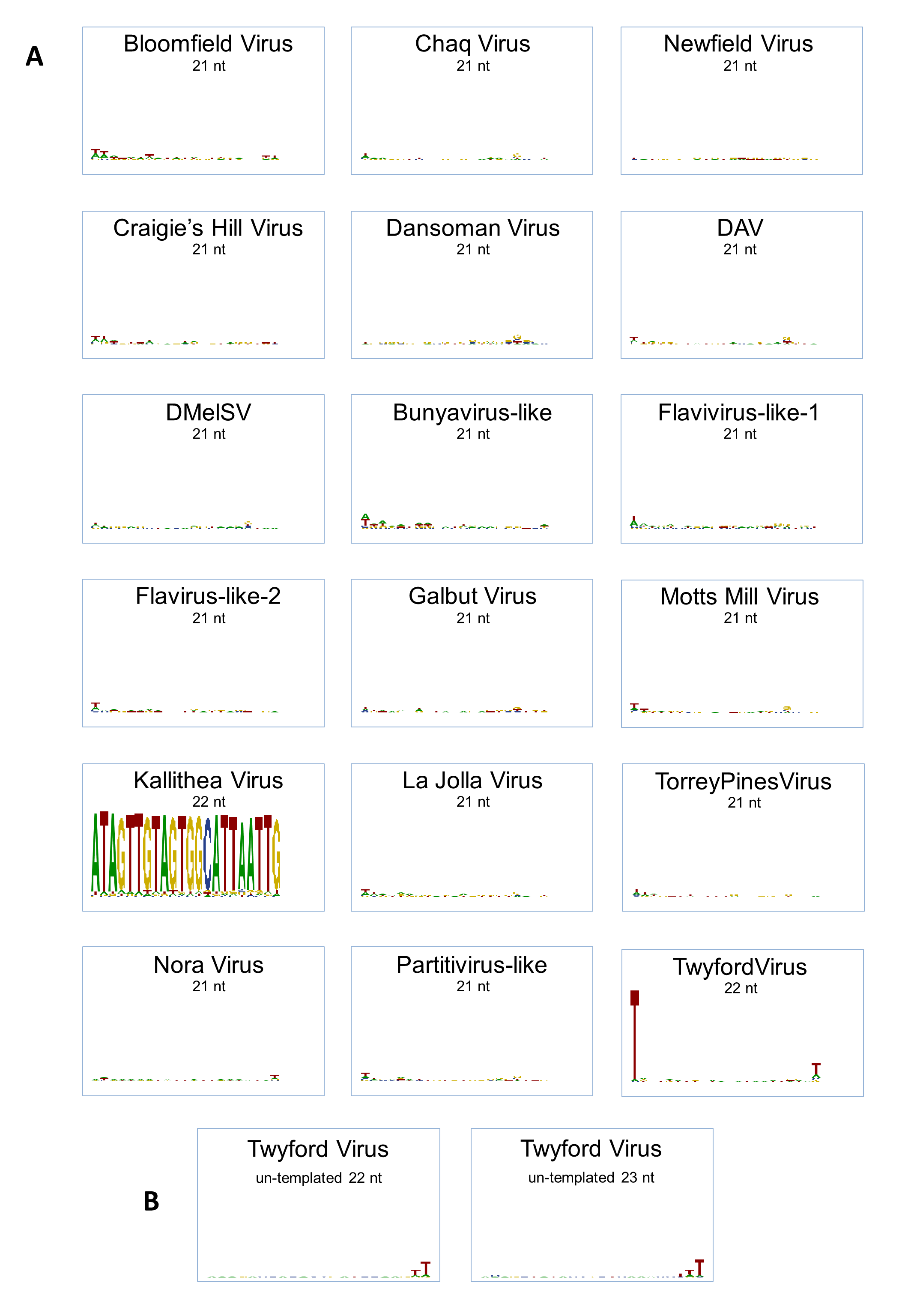

Supplement: S5 Fig — Part A “sequence logo” plots show biased base composition along the length of virus-derived small RNAs, where the relative letter sizes indicates the base frequency among reads at that position, and the total height represents the information content (which quantifies bias away from equal frequencies). For each virus, only the most common read length is shown, and reads are combined across all sequencing libraries. Note that most viruses show a small bias toward A and U (plotted as T) at the 5′ base, while Twyford Virus is biased toward U at both 5′ and 3′ positions. Kallithea DNA virus 22 nt reads are dominated by the miRNA ATAGTTGTAGTGGCATTAATTG. Part B “sequence logo” plots show biased base composition amongst viRNA-genome mismatches in 22 nt and 23 nt viRNAs from Twyford Virus (KP714075), indicating that many of the 3′U residues are non-templated. Raw count data for this figure are provided in S1 Data. (TIF) [file pbio.1002210.s014.tif]

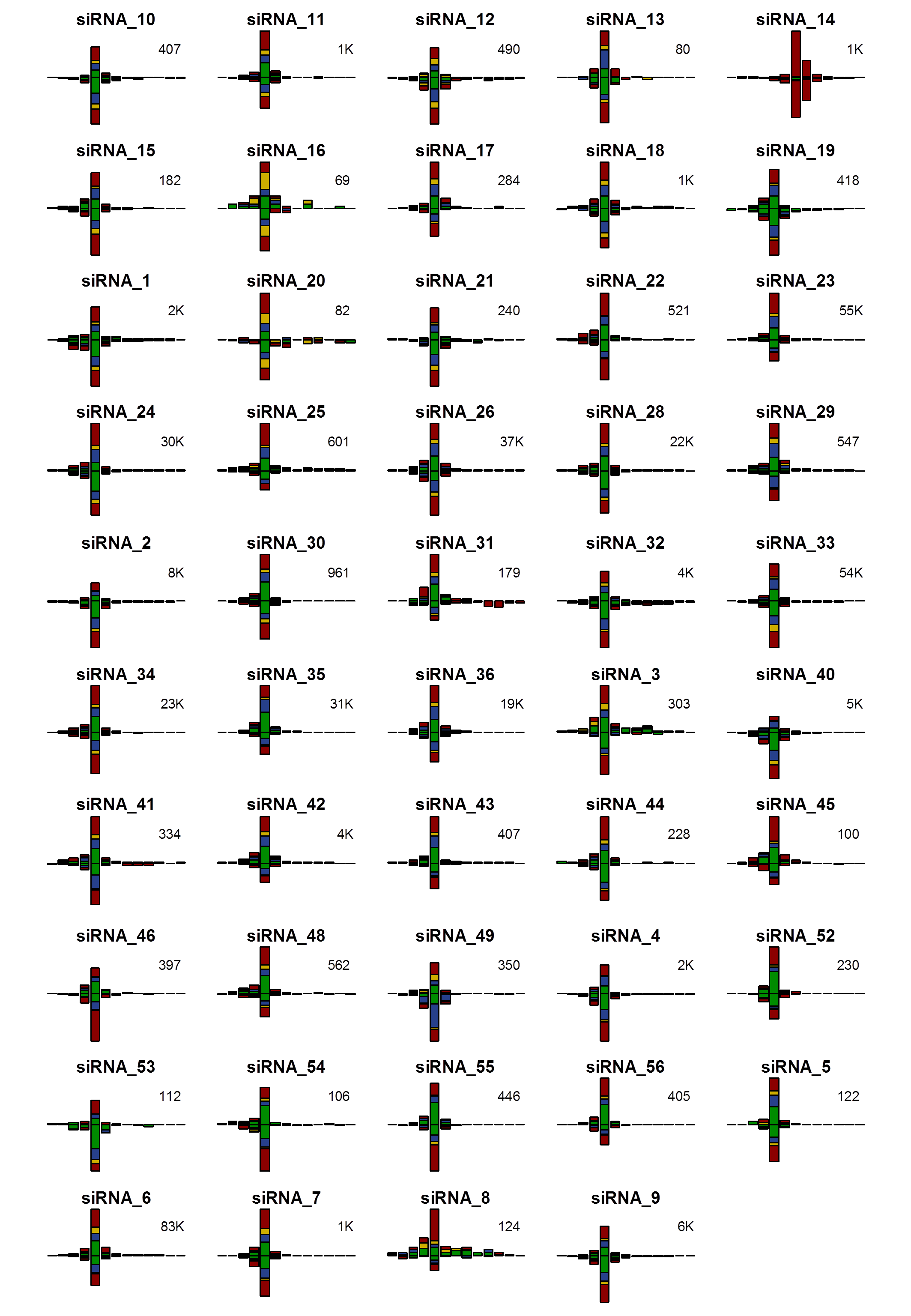

Supplement: S6 Fig — The bar plots show the size distribution of small RNAs (17–29 nt) for those unnamed siRNA-candidate loci that have >80 small RNA reads. Inset is the total number of small RNA reads summed across all libraries. Bars plotted above the x-axis represent reads mapping to the positive strand, and bars below the x-axis represent reads mapping to the negative strand. Bars are coloured according to the proportion of reads with each 5′-base (A-green, C-blue, G-yellow, U-red). All peak at 21 nt and show the expected 5′-base composition (a slight bias against G), except for siRNA Candidate 14 (KP757950) which shows the 22–23 nt 5′ U-rich peak seen in Twyford Virus (KP714075). Count data are provided in S1 Data. (TIF) [file pbio.1002210.s015.tif]

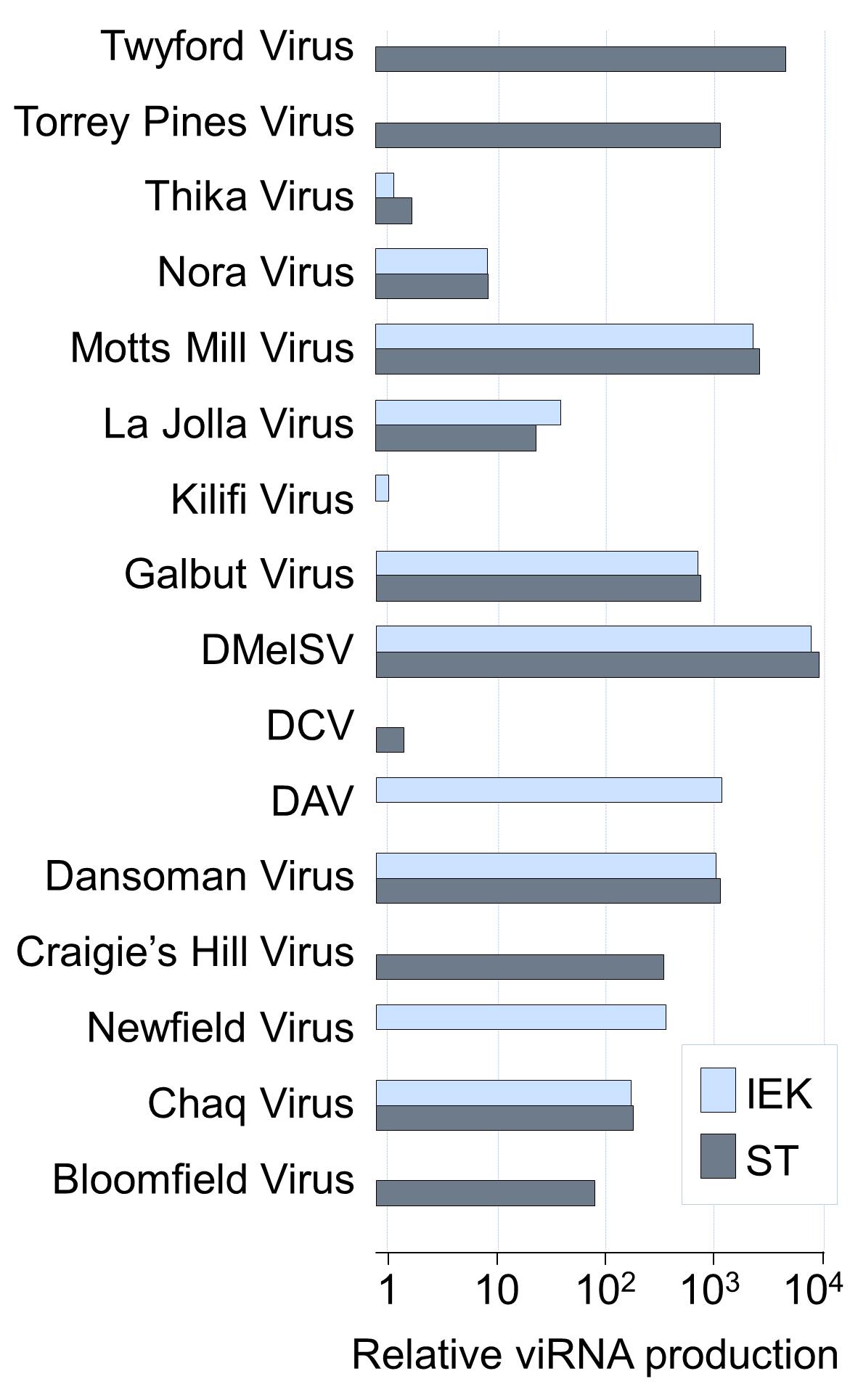

Supplement: S7 Fig — To quantify differences in the rate at which small RNAs are generated from different viruses, we calculated the relative viRNA production for each of 16 different viruses in the ST and EIK metagenomic sequencing pools. The bars show the ratio between the relative number of 21–23 nt small RNAs mapping to each virus (normalised by number of reads of the abundant Drosophila miRNA miR-34-5p), and the number of virus RNA-seq reads (relative to non-viral RNAseq reads, excluding rRNA reads). Ratios were then normalised to the lowest rate (Kilifi Virus in sample EIK) to give relative rates, such that 104-fold more viRNAs are derived from DMelSV than from Kilifi Virus. Where viruses were present in both the EIK and ST pools, the correlation between the two datasets is high (rank correlation coefficient >0.99), suggesting that this variation is repeatable. Normalised data are provided in S1 Data. (TIF) [file pbio.1002210.s016.tif]

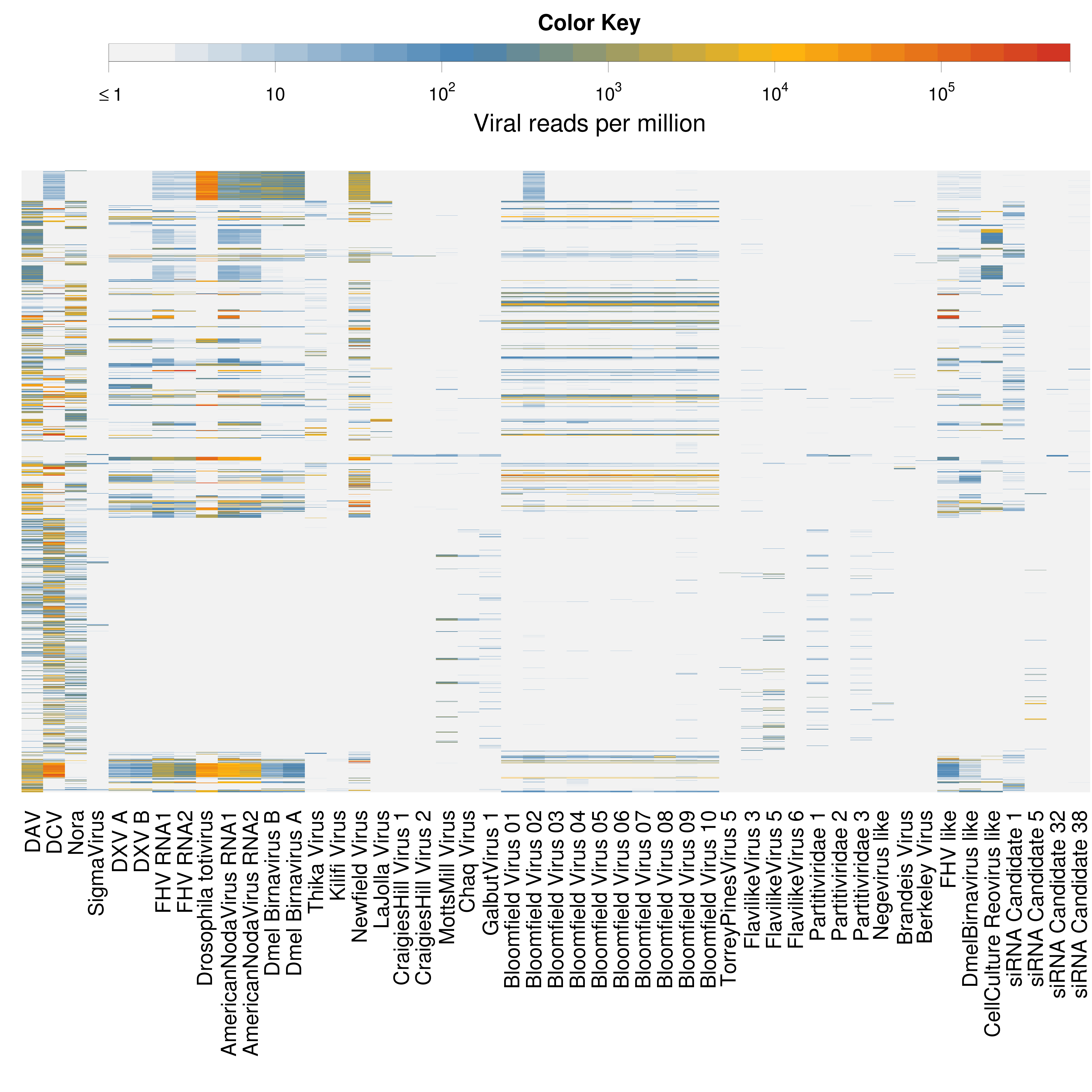

Supplement: S8 Fig — The grid shows the proportion of reads from each of 3,144 publicly available RNAseq and small RNA datasets from Drosophila and Drosophila cell culture (vertical axis) that map to viruses (horizontal axis). Only datasets that have at least one virus present at ≥100 viral reads per million total reads were included, and only viruses present in at least one dataset at ≥100 viral reads per million total reads were included. Note that different parts of the segmented viruses generally co-occur within datasets, which allowed us to provisionally associate siRNA-candidate sequences with BLAST-candidate sequences (e.g., Nodaviruses and Bloomfield Virus). The viruses from DAV to Drosophila melanogaster Birnavirus were reported previously, the others are newly described here. Counts are provided in S5 Data. (TIFF) [file pbio.1002210.s017.tiff]

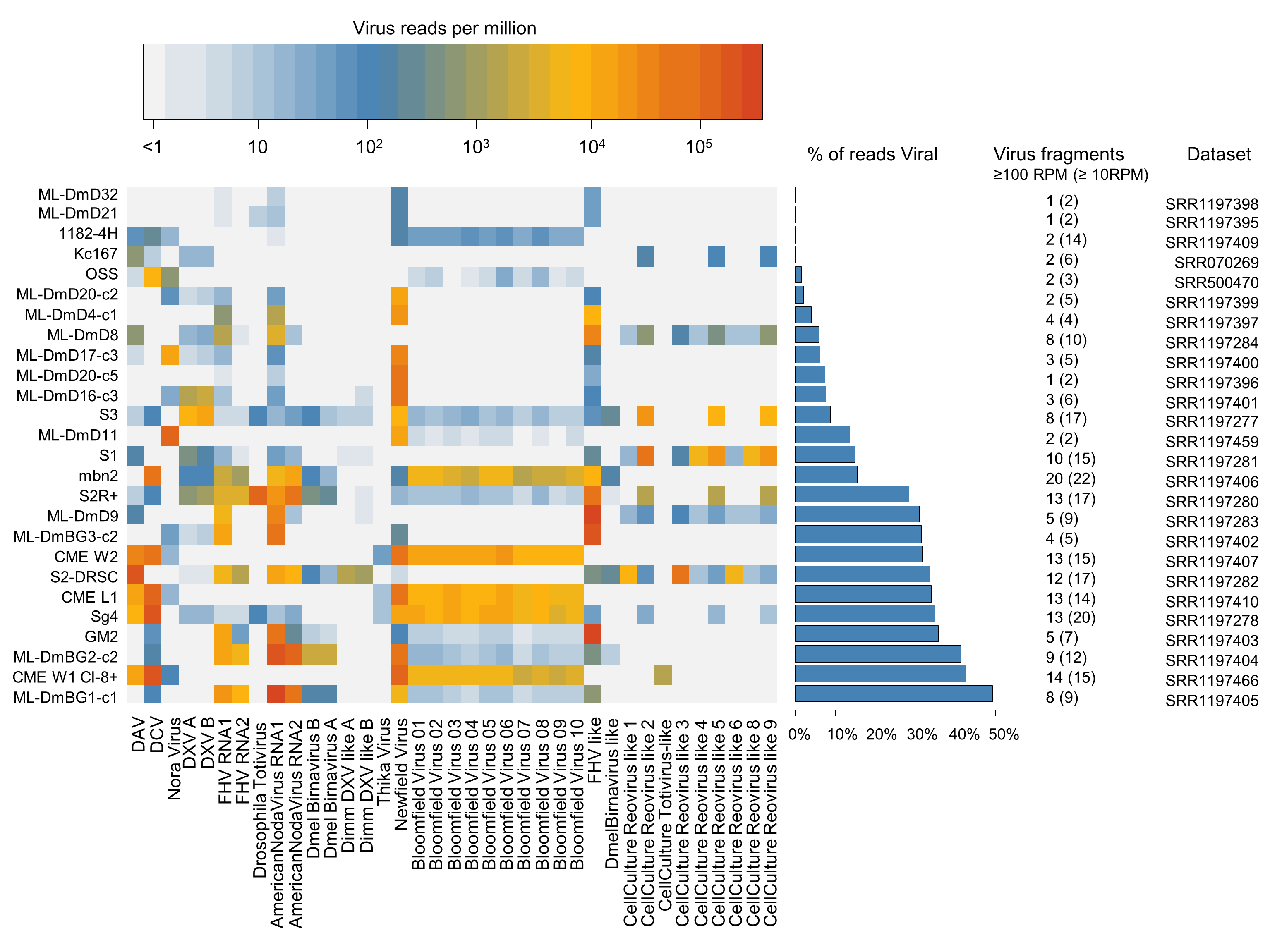

Supplement: S9 Fig — A single exemplar RNAseq dataset was selected for each of 26 widely-used Drosophila cell culture lines, and all forward reads were mapped to viruses. Twenty-four of the datasets were drawn from ModEncode cell culture sequencing [82], and two that were not available (OSS and Kc167) were taken from datasets SRR070269 and SRR500470. The colour scale illustrates the fraction of reads that were viral in origin (virus reads per million total reads) and viruses are sorted in ascending order of viral read fraction (from ML-DmD32 with <1% viral reads, to ML-DmBG1-c1 with approximately 50% viral reads). Note that the virus population is likely to differ between different subcultures, and these values should only be considered illustrative. Counts are provided in S5 Data. (TIF) [file pbio.1002210.s018.tif]

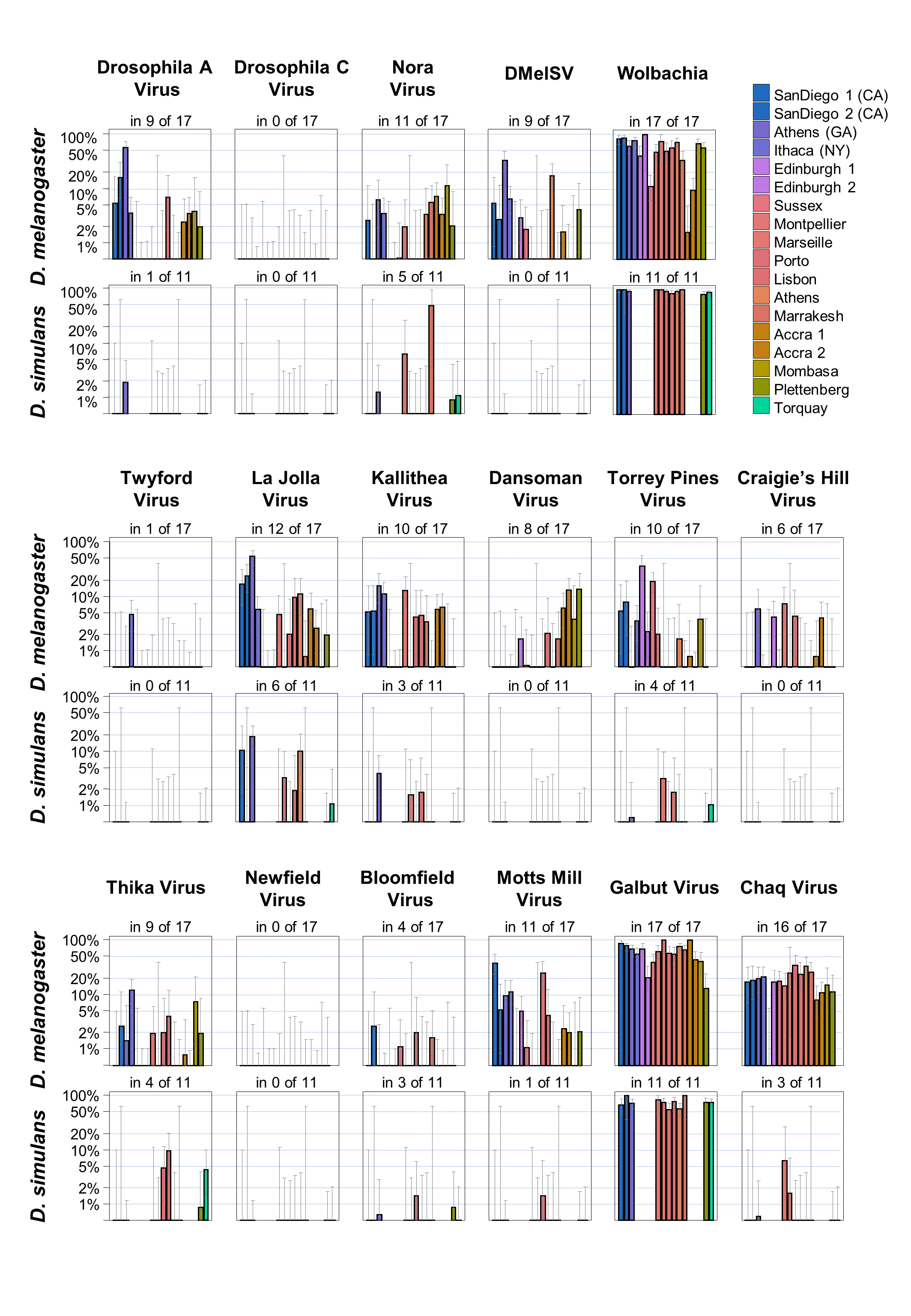

Supplement: S10 Fig — Charts show virus and Wolbachia prevalence in D. melanogaster and D. simulans for each of the 17 locations at which flies were collected. Values are maximum likelihood estimates with 2 log-likelihood intervals, and are plotted on a log scale. Where no bar or confidence interval is provided, no flies of that species were sampled. Location details and population prevalence are provided in S4 Table. (TIF) [file pbio.1002210.s019.tif]

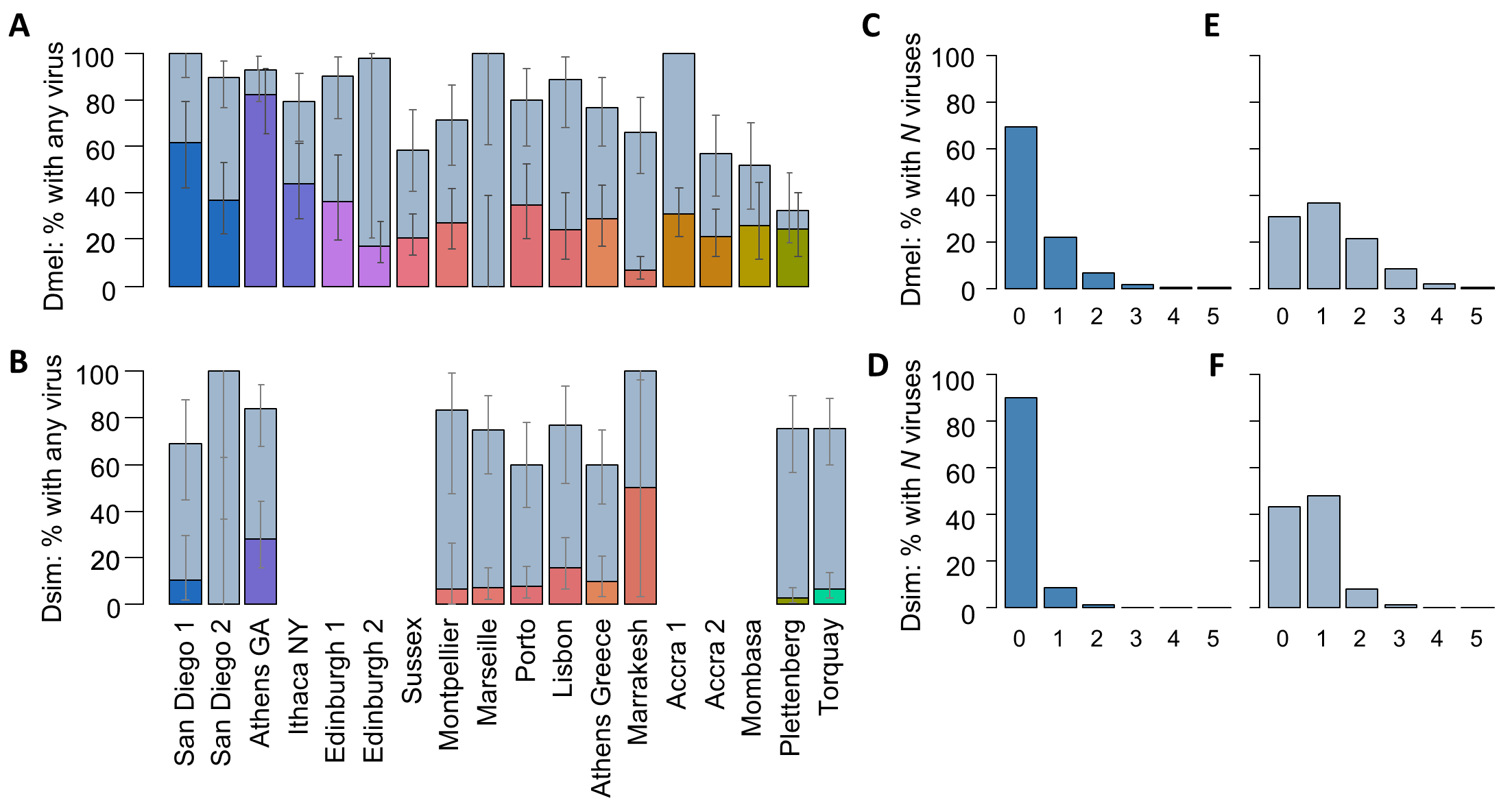

Supplement: S11 Fig — Panels (A) and (B) show the percentage of D. melanogaster and D. simulans carrying at least one of the surveyed viruses. Bars are maximum-likelihood estimates with 2 log-likelihood intervals. Coloured bars represent carriers of DAV, DCV, Nora, DMelSV, Twyford, La Jolla, Kallithea, Dansoman, Torrey Pines, Craigie’s Hill, Thika, Newfield, Bloomfield, or Motts Mill viruses (but excluding the high-prevalence siRNA-candidate viruses); grey bars show the large increase in overall prevalence if the siRNA-candidate viruses (Galbut Virus and Chaq Virus) are included. Missing bars indicate that the host species was absent from the collection location (for collection details and prevalence, see S4 Table). Panels (C) and (E) show the proportion of virus-free and multiply-infected D. melanogaster when Galbut Virus and Chaq Virus are excluded (C) or included (E), while (D) and (F) show the equivalent plots for D. simulans. Panels (C-F) are calculated using single-fly assays only (not bulks), and are averages across populations. (TIF) [file pbio.1002210.s020.tif]

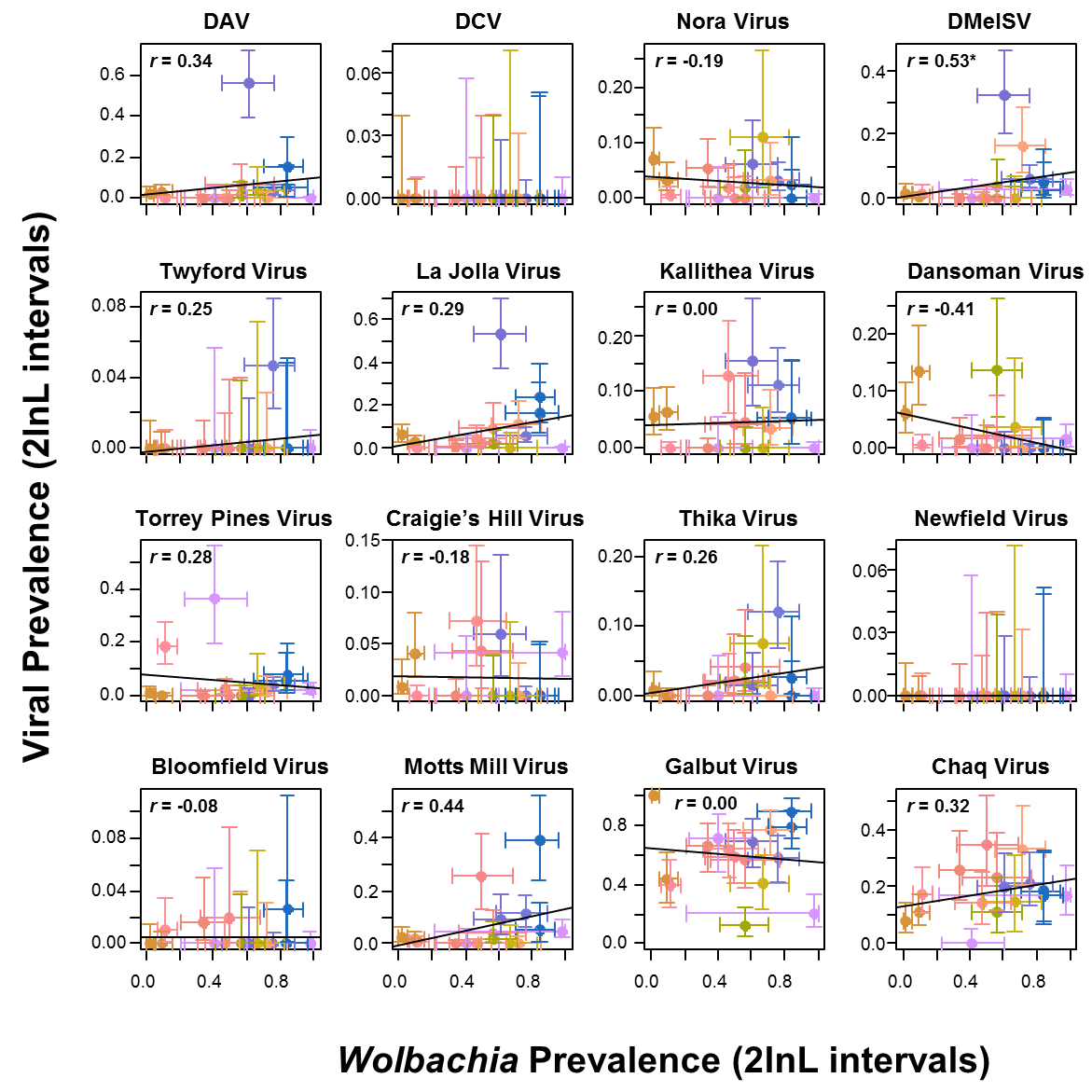

Supplement: S12 Fig — Each plot shows the correlation between the named virus and Wolbachia in prevalence, across the sampled populations. Inset are rank correlation coefficients, and a simple linear regression (virus ~ Wolbachia) for illustration. The analysis does not account for any spatial autocorrelation in prevalence, and does not correct for multiple testing. Points are coloured by location (see S10 and S11 Figs for colours) and plotted with 2 log-likelihood intervals. Nominal “significance” at p < 0.05 is shown using asterisks. Values are provided in S4 Table. (TIF) [file pbio.1002210.s021.tif]

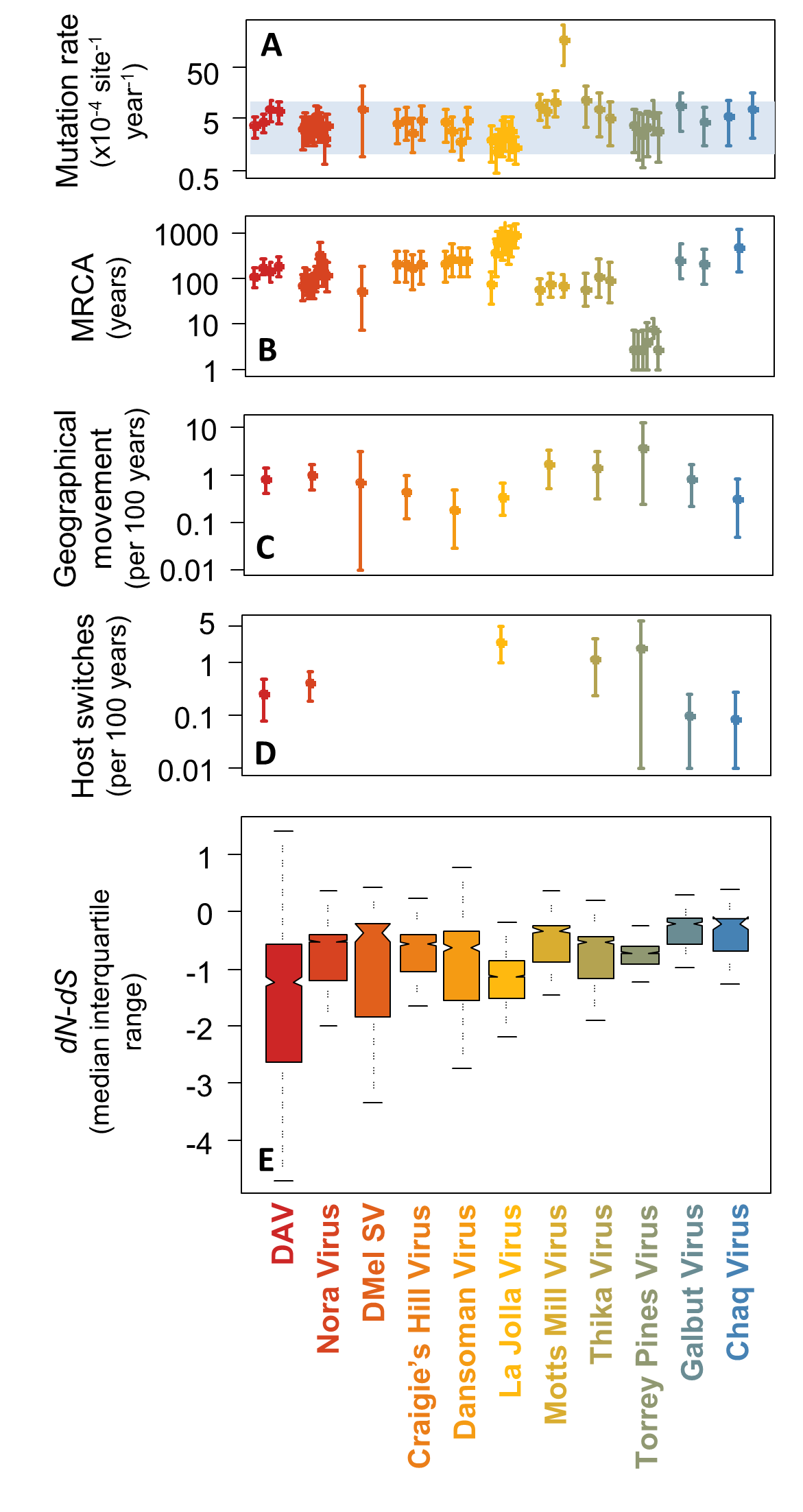

Supplement: S13 Fig — Each panel shows parameter estimates of viral evolution for the eleven RNA viruses for which sufficient sequence data were available. Panels are (A) mutation rate, (B) date for the most recent common ancestor, (C) the range of movement between geographic regions (defined as continents and laboratory), (D) the rate of switching between D. melanogaster and D. simulans, and (E) the relative rates of synonymous (dS) and nonsynonymous (dN) substitution (dN-dS > 0 implies positive selection). For panels A–D points are the median of the posterior sample and 95% credibility intervals, panel E shows the median and range across codons. For all viruses except DAV and Nora Virus, a strong prior was placed on mutation rate (Panel A, grey box), and mutation rates and MRCA dates were inferred separately for each alignment block. The underlying BEAST XML files (including alignments and model specifications) are provided, along with the resulting mcc tree files and summaries of posterior distributions, in S7 Data and S8 Data. The underlying FUBAR batch files and per-site parameter estimates are provided in S9 Data. (TIF) [file pbio.1002210.s022.tif]

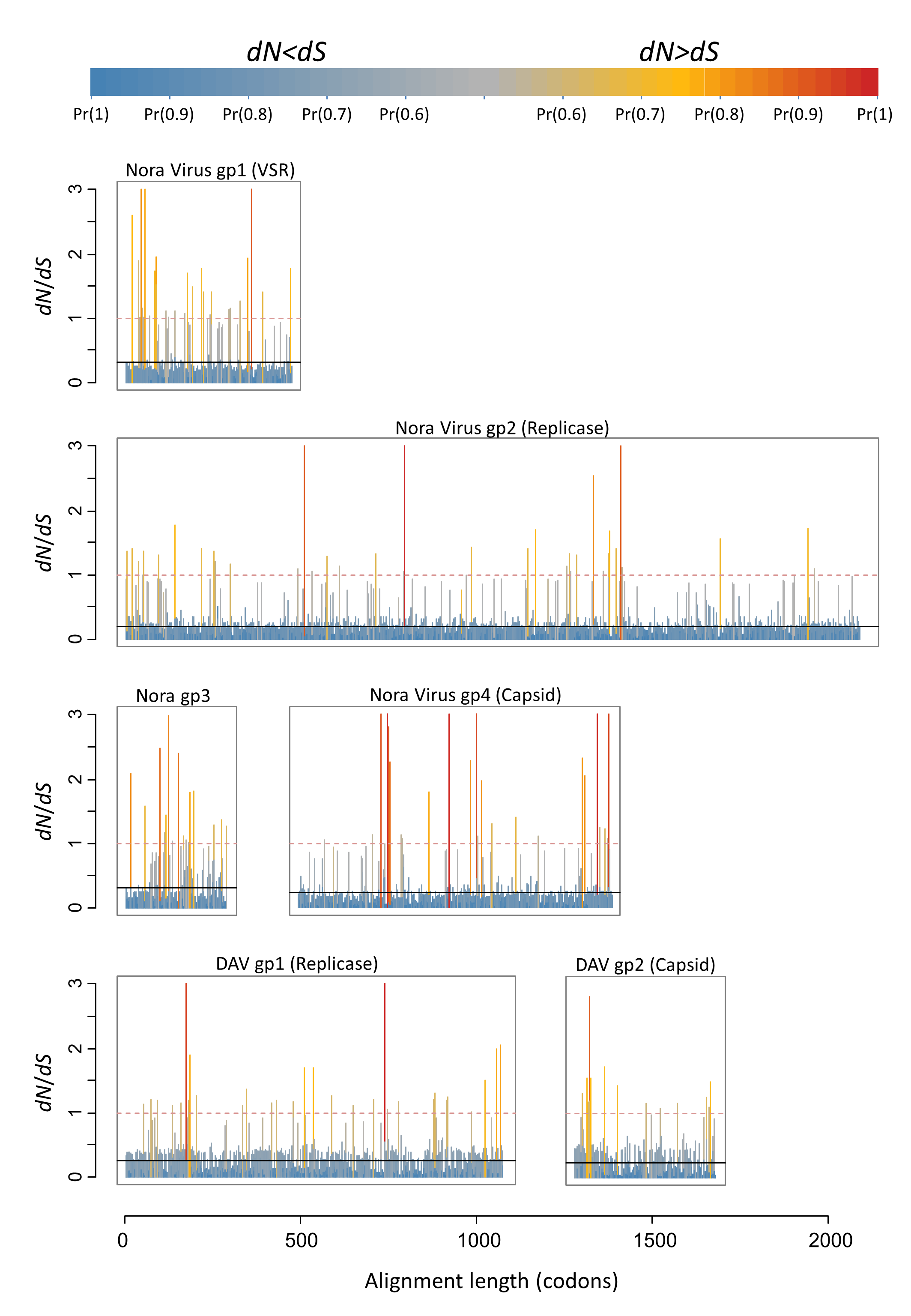

Supplement: S14 Fig — Point estimates of dN/dS are shown for each codon in open reading frames of DAV and Nora Virus (ratios of the posterior estimates, not the posterior estimates of the ratios). Bar colours illustrate posterior support that the site is constrained (blue for strong support that dN < dS) or positively selected (red for strong support that dN > dS). Positions coloured grey have little support for either positive selection or constraint. The dashed horizontal line indicates neutrality (dN = dS), so that bars higher than this are candidate sites for positive selection. The solid horizontal line shows the mean of the dN/dS estimates for that open reading frame. dN/dS estimates greater than 3 have been truncated to 3 for clarity. FUBAR batch files and parameter estimates are provided in S9 Data. (TIF) [file pbio.1002210.s023.tif]
