# Supplementary material for: The Discovery, Distribution, and Evolution of Viruses Associated with Drosophila melanogaster
Source: PLoS Biol. 2015 Jul 14;13(7):e1002210. doi: 10.1371/journal.pbio.1002210 (PMC4501690; doi:10.1371/journal.pbio.1002210)
Supplement: S3 Fig — Mid-point rooted maximum clade-credibility trees showing the inferred relationship between new and previously known Drosophila–associated viruses (red), previously published viruses from other taxa (black) and sequences from the Transcriptome Shotgun Assemblies (blue). Bayesian posterior support is shown for second-order nodes above and where space permits, and the scale is given in amino-acid substitutions per site. Genbank nucleic acid or protein identifiers are given after each sequence. (A) Craigie’s Hill Virus and Nodaviruses; (B) Kilifi Virus, Thika Virus, DCV, and Dicistroviruses; (C) Bloomfield Virus, Torrey Pines Virus, and Reoviruses; (D) Newfield Virus, DAV, and Permutotetraviruses; (E) Galbut Virus and TSA sequences; (F) La Jolla Virus, Twyford Virus, and a closely related Iflaviruses; (G) Motts Mill Virus, Sobemoviruses, and Poleroviruses; (H) Charvil Virus and Flaviviruses; (I) Dansoman Virus and viruses related to Chronic Bee Paralysis Virus; (J) Chaq Virus and TSA sequences; (K) Brandeis Virus and related Negeviruses; (L) Berkeley Virus, DCV, and a selection of Picornavirales sequences; (M) Partitiviruses; (N) Bunyaviruses; (O) Kallithea Virus and Nudiviruses (Note that D. innubila Nudivirus [56] is excluded because the relevant loci are unavailable). Alignments are provided in S3 Data, and maximum clade credibility trees are provided in S4 Data. (PDF) [file pbio.1002210.s012.pdf]

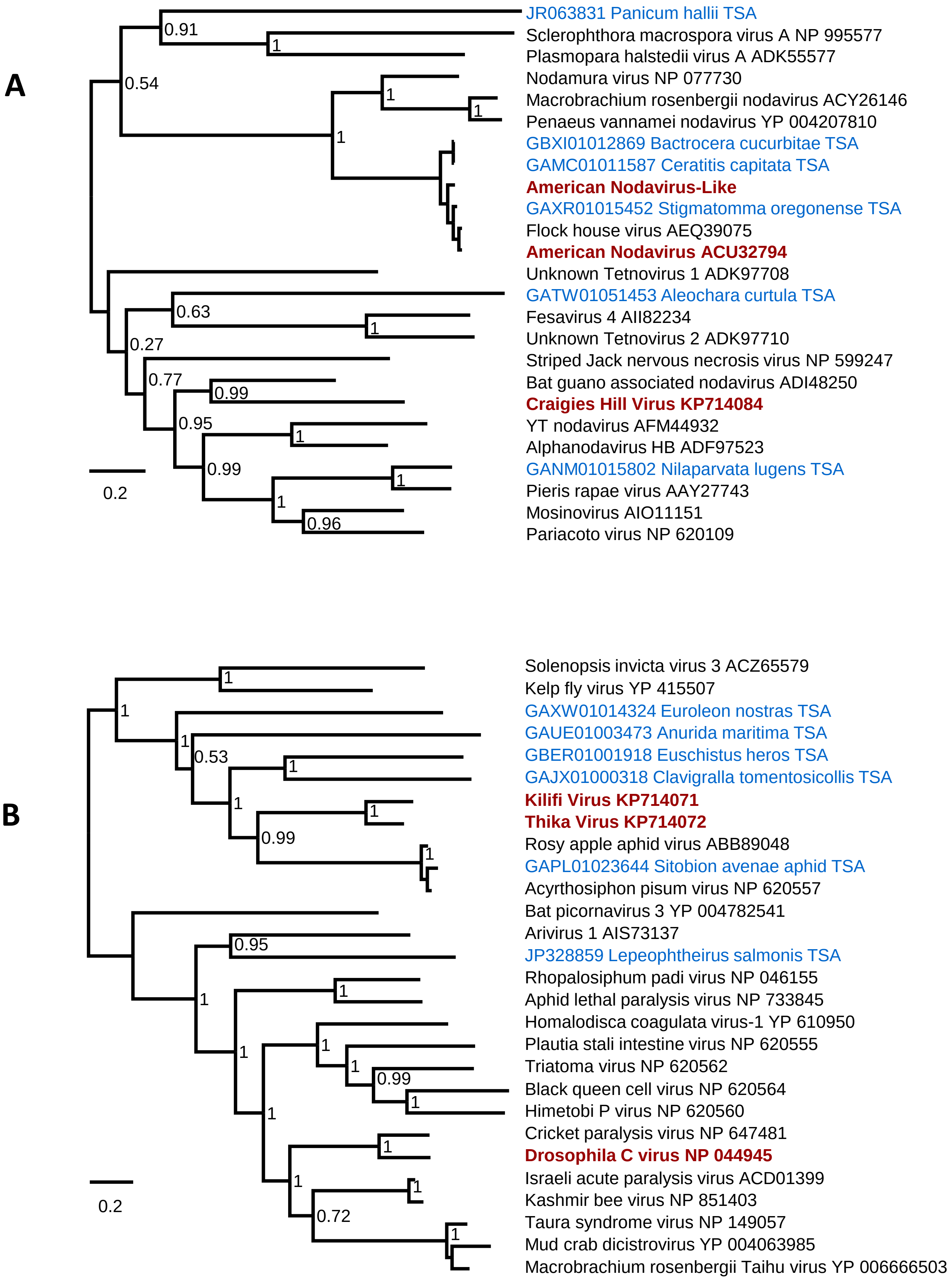
A
JR063831 Panicum hallii TSA
Sclerophthora macrospora virus A NP 995577
Plasmopara halstedii virus A ADK55577
Nodamura virus NP 077730
Macrobrachium rosenbergii nodavirus ACY26146
Penaeus vannamei nodavirus YP 004207810
GBXI01012869 Bactrocera cucurbitae TSA
GAMC01011587 Ceratitis capitata TSA
American Nodavirus-Like
GAXR01015452 Stigmatomma oregonense TSA
Flock house virus AEQ39075
American Nodavirus ACU32794
Unknown Tetnovirus 1 ADK97708
GATW01051453 Aleochara curtula TSA
Fesavirus 4 AII82234
Unknown Tetnovirus 2 ADK97710
Striped Jack nervous necrosis virus NP 599247
Bat guano associated nodavirus ADI48250
Craigies Hill Virus KP714084
YT nodavirus AFM44932
Alphanodavirus HB ADF97523
GANM01015802 Nilaparvata lugens TSA
Pieris rapae virus AAY27743
Mosinovirus AIO11151
Pariacoto virus NP 620109
0.2
B
Solenopsis invicta virus 3 ACZ65579
Kelp fly virus YP 415507
GAXW01014324 Euroleon nostras TSA
GAUE01003473 Anurida maritima TSA
GBER01001918 Euschistus heros TSA
GAJX01000318 Clavigralla tomentosicollis TSA
Kilifi Virus KP714071
Thika Virus KP714072
Rosy apple aphid virus ABB89048
GAPL01023644 Sitobion avenae aphid TSA
Acyrthosiphon pisum virus NP 620557
Bat picornavirus 3 YP 004782541
Arivirus 1 AIS73137
JP328859 Lepeophtheirus salmonis TSA
Rhopalosiphum padi virus NP 046155
Aphid lethal paralysis virus NP 733845
Homalodisca coagulata virus-1 YP 610950
Plautia stali intestine virus NP 620555
Triatoma virus NP 620562
Black queen cell virus NP 620564
Himetobi P virus NP 620560
Cricket paralysis virus NP 647481
Drosophila C virus NP 044945
Israeli acute paralysis virus ACD01399
Kashmir bee virus NP 851403
Taura syndrome virus NP 149057
Mud crab dicistrovirus YP 004063985
Macrobrachium rosenbergii Taihu virus YP 006666503
0.2

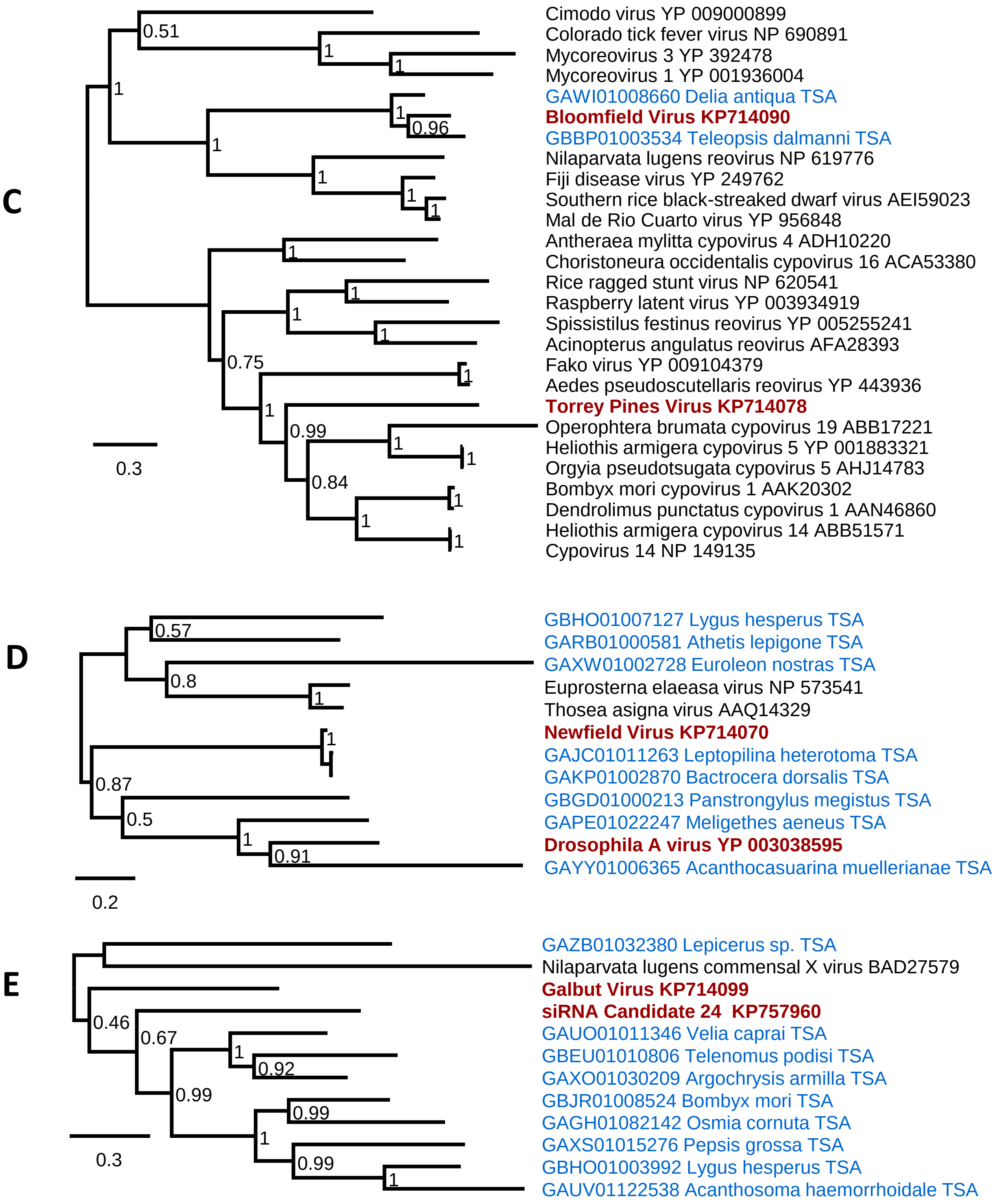
C
Cimodo virus YP 009000899
Colorado tick fever virus NP 690891
Mycoreovirus 3 YP 392478
Mycoreovirus 1 YP 001936004
GAWI01008660 Delia antiqua TSA
Bloomfield Virus KP714090
GBBP01003534 Teleopsis dalmanni TSA
Nilaparvata lugens reovirus NP 619776
Fiji disease virus YP 249762
Southern rice black-streaked dwarf virus AEI59023
Mal de Rio Cuarto virus YP 956848
Antheraea mylitta cypovirus 4 ADH10220
Choristoneura occidentalis cypovirus 16 ACA53380
Rice ragged stunt virus NP 620541
Raspberry latent virus YP 003934919
Spissistilus festinus reovirus YP 005255241
Acinopterus angulatus reovirus AFA28393
Fako virus YP 009104379
Aedes pseudoscutellaris reovirus YP 443936
Torrey Pines Virus KP714078
Operophtera brumata cypovirus 19 ABB17221
Heliothis armigera cypovirus 5 YP 001883321
Orgyia pseudotsugata cypovirus 5 AHJ14783
Bombyx mori cypovirus 1 AAK20302
Dendrolimus punctatus cypovirus 1 AAN46860
Heliothis armigera cypovirus 14 ABB51571
Cypovirus 14 NP 149135
0.51
1
0.96
0.75
0.99
0.84
0.3
D
GBHO01007127 Lygus hesperus TSA
GARB01000581 Athetis lepigone TSA
GAXW01002728 Euroleon nostras TSA
Euprosterna elaeasa virus NP 573541
Thosea asigna virus AAQ14329
Newfield Virus KP714070
GAJC01011263 Leptopilina heterotoma TSA
GAKP01002870 Bactrocera dorsalis TSA
GBGD01000213 Panstrongylus megistus TSA
GAPE01022247 Meligethes aeneus TSA
Drosophila A virus YP 003038595
GAYY01006365 Acanthocasuarina muellerianae TSA
0.57
0.8
0.87
0.5
0.91
0.2
E
GAZB01032380 Lepicerus sp. TSA
Nilaparvata lugens commensal X virus BAD27579
Galbut Virus KP714099
siRNA Candidate 24 KP757960
GAUO01011346 Velia caprai TSA
GBEU01010806 Telenomus podisi TSA
GAXO01030209 Argochrysis armilla TSA
GBJR01008524 Bombyx mori TSA
GAGH01082142 Osmia cornuta TSA
GAXS01015276 Pepsis grossa TSA
GBHO01003992 Lygus hesperus TSA
GAUV01122538 Acanthosoma haemorrhoidale TSA
0.46
0.67
0.92
0.99
0.3

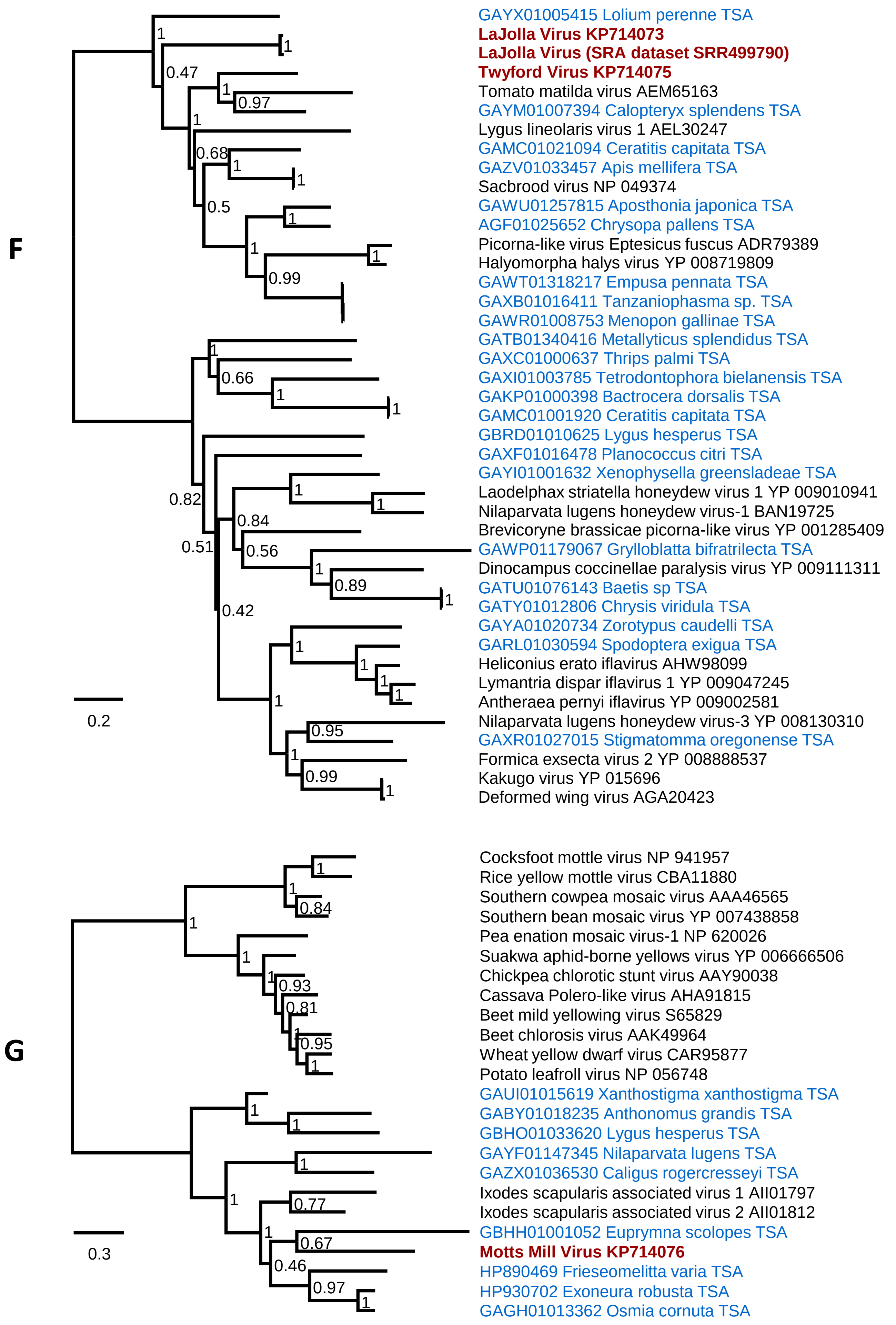
F
GAYX01005415 Lolium perenne TSA
LaJolla Virus KP714073
LaJolla Virus (SRA dataset SRR499790)
Twyford Virus KP714075
Tomato matilda virus AEM65163
GAYM01007394 Calopteryx splendens TSA
Lygus lineolaris virus 1 AEL30247
GAMC01021094 Ceratitis capitata TSA
GAZV01033457 Apis mellifera TSA
Sacbrood virus NP 049374
GAWU01257815 Aposthonia japonica TSA
AGF01025652 Chrysopa pallens TSA
Picorna-like virus Eptesicus fuscus ADR79389
Halyomorpha halys virus YP 008719809
GAWT01318217 Empusa pennata TSA
GAXB01016411 Tanzaniophasma sp. TSA
GAWR01008753 Menopon gallinae TSA
GATB01340416 Metallyticus splendidus TSA
GAXC01000637 Thrips palmi TSA
GAXI01003785 Tetrodontophora bielanensis TSA
GAKP01000398 Bactrocera dorsalis TSA
GAMC01001920 Ceratitis capitata TSA
GBRD01010625 Lygus hesperus TSA
GAXF01016478 Planococcus citri TSA
GAYI01001632 Xenophysella greensladeae TSA
Laodelphax striatella honeydew virus 1 YP 009010941
Nilaparvata lugens honeydew virus-1 BAN19725
Brevicoryne brassicae picorna-like virus YP 001285409
GAWP01179067 Grylloblatta bifratrilecta TSA
Dinocampus coccinellae paralysis virus YP 009111311
GATU01076143 Baetis sp TSA
GATY01012806 Chrysis viridula TSA
GAYA01020734 Zorotypus caudelli TSA
GARL01030594 Spodoptera exigua TSA
Heliconius erato iflavirus AHW98099
Lymantria dispar iflavirus 1 YP 009047245
Antheraea pernyi iflavirus YP 009002581
Nilaparvata lugens honeydew virus-3 YP 008130310
GAXR01027015 Stigmatomma oregonense TSA
Formica exsecta virus 2 YP 008888537
Kakugo virus YP 015696
Deformed wing virus AGA20423
1
1
0.47
1
0.97
1
0.68
1
1
0.5
1
1
1
0.99
1
0.66
1
1
0.82
0.51
1
1
0.84
0.56
1
0.89
1
0.42
1
1
1
1
1
0.95
1
0.99
1
0.2
G
Cocksfoot mottle virus NP 941957
Rice yellow mottle virus CBA11880
Southern cowpea mosaic virus AAA46565
Southern bean mosaic virus YP 007438858
Pea enation mosaic virus-1 NP 620026
Suakwa aphid-borne yellows virus YP 006666506
Chickpea chlorotic stunt virus AAY90038
Cassava Polero-like virus AHA91815
Beet mild yellowing virus S65829
Beet chlorosis virus AAK49964
Wheat yellow dwarf virus CAR95877
Potato leafroll virus NP 056748
GAUI01015619 Xanthostigma xanthostigma TSA
GABY01018235 Anthonomus grandis TSA
GBHO01033620 Lygus hesperus TSA
GAYF01147345 Nilaparvata lugens TSA
GAZX01036530 Caligus rogercresseyi TSA
Ixodes scapularis associated virus 1 AII01797
Ixodes scapularis associated virus 2 AII01812
GBHH01001052 Euprymna scolopes TSA
Motts Mill Virus KP714076
HP890469 Frieseomelitta varia TSA
HP930702 Exoneura robusta TSA
GAGH01013362 Osmia cornuta TSA
1
1
0.84
1
1
1
0.93
0.81
1
0.95
1
1
1
1
1
0.77
1
0.67
0.46
0.97
1
0.3

H

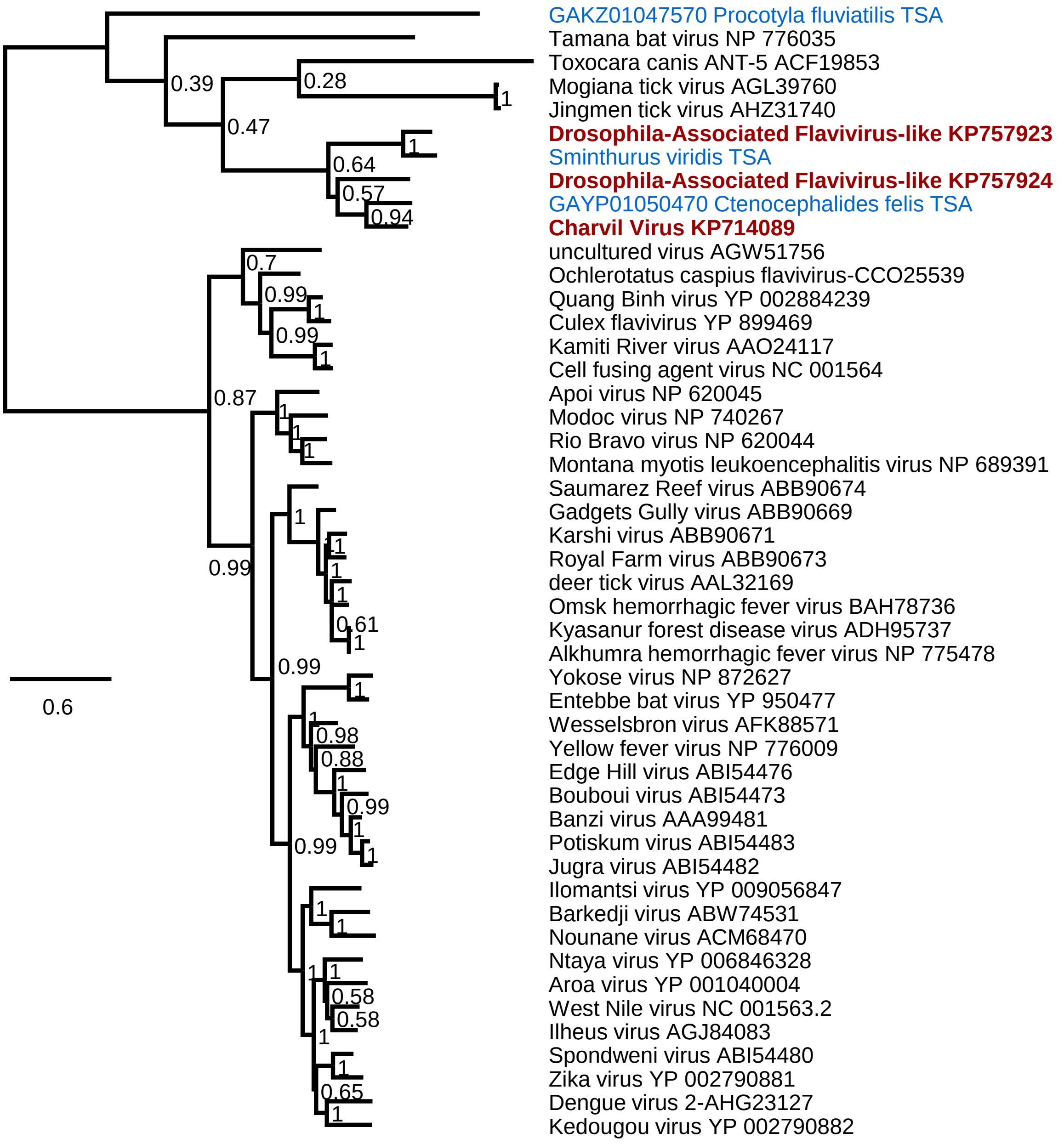
GAKZ01047570 Procotyla fluviatilis TSA
Tamana bat virus NP 776035
Toxocara canis ANT-5 ACF19853
Mogiana tick virus AGL39760
Jingmen tick virus AHZ31740
Drosophila-Associated Flavivirus-like KP757923
Sminthurus viridis TSA
Drosophila-Associated Flavivirus-like KP757924
GAYP01050470 Ctenocephalides felis TSA
Charvil Virus KP714089
uncultured virus AGW51756
Ochlerotatus caspius flavivirus-CCO25539
Quang Binh virus YP 002884239
Culex flavivirus YP 899469
Kamiti River virus AAO24117
Cell fusing agent virus NC 001564
Apoi virus NP 620045
Modoc virus NP 740267
Rio Bravo virus NP 620044
Montana myotis leukoencephalitis virus NP 689391
Saumarez Reef virus ABB90674
Gadgets Gully virus ABB90669
Karshi virus ABB90671
Royal Farm virus ABB90673
deer tick virus AAL32169
Omsk hemorrhagic fever virus BAH78736
Kyasanur forest disease virus ADH95737
Alkhumra hemorrhagic fever virus NP 775478
Yokose virus NP 872627
Entebbe bat virus YP 950477
Wesselsbron virus AFK88571
Yellow fever virus NP 776009
Edge Hill virus ABI54476
Bouboui virus ABI54473
Banzi virus AAA99481
Potiskum virus ABI54483
Jugra virus ABI54482
Ilomantsi virus YP 009056847
Barkedji virus ABW74531
Nounane virus ACM68470
Ntaya virus YP 006846328
Aroa virus YP 001040004
West Nile virus NC 001563.2
Ilheus virus AGJ84083
Spondweni virus ABI54480
Zika virus YP 002790881
Dengue virus 2-AHG23127
Kedougou virus YP 002790882
0.39
0.28
0.47
0.64
0.57
0.94
0.7
0.99
0.99
0.87
0.99
0.61
0.99
0.98
0.88
0.99
0.99
0.58
0.58
0.65
0.6

I

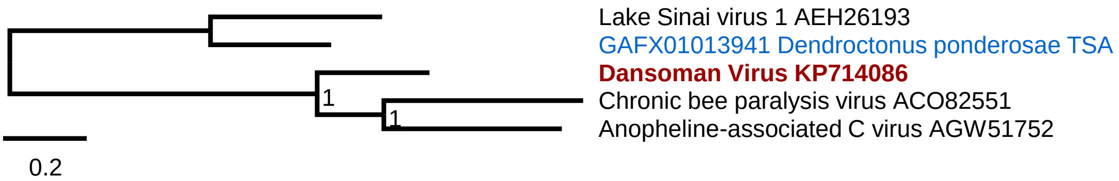
Lake Sinai virus 1 AEH26193
GAFX01013941 Dendroctonus ponderosae TSA
Dansoman Virus KP714086
Chronic bee paralysis virus ACO82551
Anopheline-associated C virus AGW51752
1
1
0.2

J

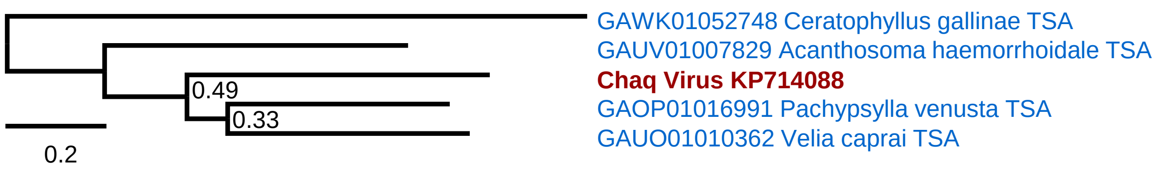
GAWK01052748 Ceratophyllus gallinae TSA
GAUV01007829 Acanthosoma haemorrhoidale TSA
Chaq Virus KP714088
GAOP01016991 Pachypsylla venusta TSA
GAUO01010362 Velia caprai TSA
0.49
0.33
0.2

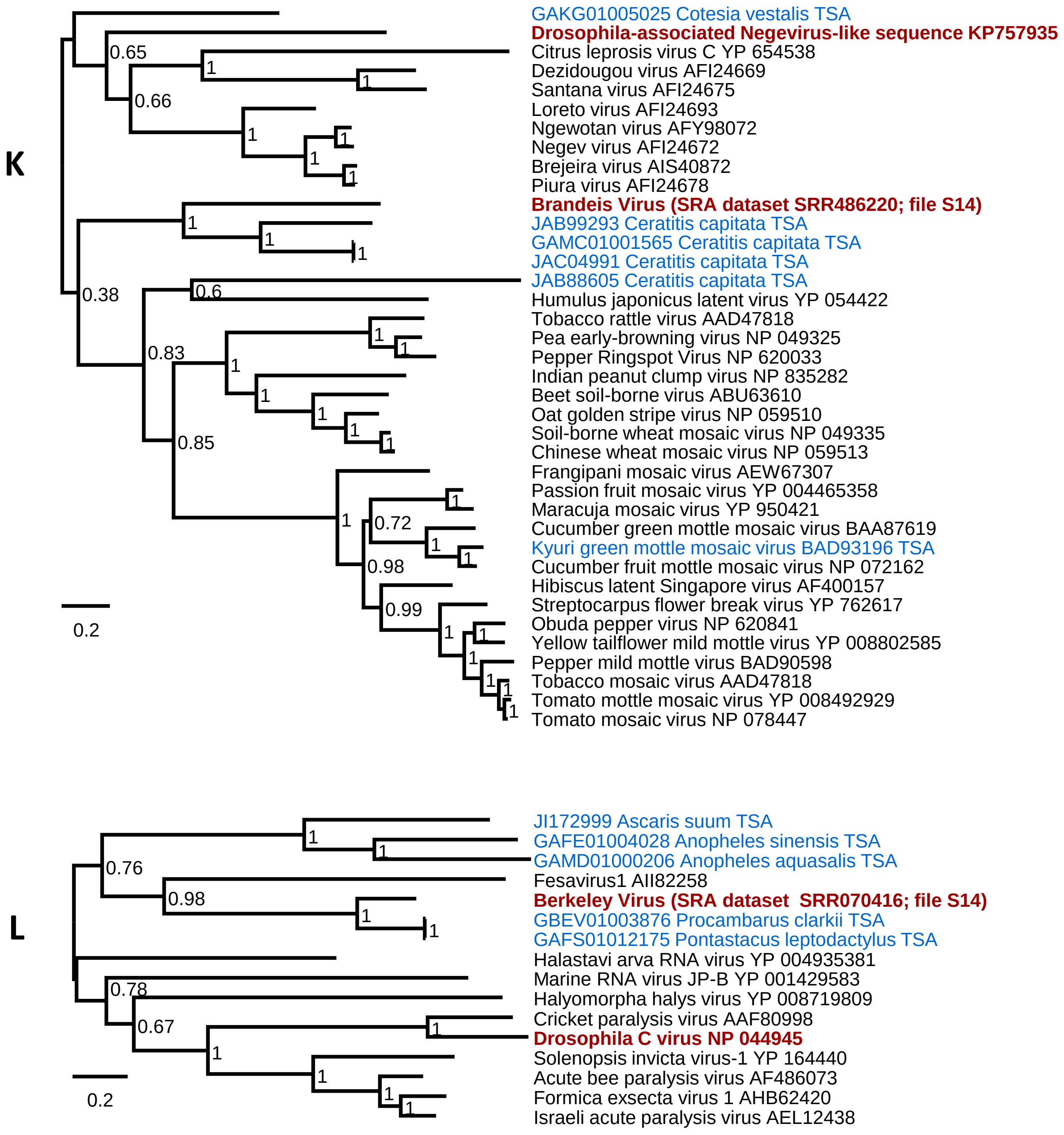
K
GAKG01005025 Cotesia vestalis TSA
Drosophila-associated Negevirus-like sequence KP757935
Citrus leprosis virus C YP 654538
Dezidougou virus AFI24669
Santana virus AFI24675
Loreto virus AFI24693
Ngewotan virus AFY98072
Negev virus AFI24672
Brejeira virus AIS40872
Piura virus AFI24678
Brandeis Virus (SRA dataset SRR486220; file S14)
JAB99293 Ceratitis capitata TSA
GAMC01001565 Ceratitis capitata TSA
JAC04991 Ceratitis capitata TSA
JAB88605 Ceratitis capitata TSA
Humulus japonicus latent virus YP 054422
Tobacco rattle virus AAD47818
Pea early-browning virus NP 049325
Pepper Ringspot Virus NP 620033
Indian peanut clump virus NP 835282
Beet soil-borne virus ABU63610
Oat golden stripe virus NP 059510
Soil-borne wheat mosaic virus NP 049335
Chinese wheat mosaic virus NP 059513
Frangipani mosaic virus AEW67307
Passion fruit mosaic virus YP 004465358
Maracuja mosaic virus YP 950421
Cucumber green mottle mosaic virus BAA87619
Kyuri green mottle mosaic virus BAD93196 TSA
Cucumber fruit mottle mosaic virus NP 072162
Hibiscus latent Singapore virus AF400157
Streptocarpus flower break virus YP 762617
Obuda pepper virus NP 620841
Yellow tailflower mild mottle virus YP 008802585
Pepper mild mottle virus BAD90598
Tobacco mosaic virus AAD47818
Tomato mottle mosaic virus YP 008492929
Tomato mosaic virus NP 078447
0.65
0.66
0.38
0.6
0.83
0.85
0.72
0.98
0.99
0.2
L
JI172999 Ascaris suum TSA
GAFE01004028 Anopheles sinensis TSA
GAMD01000206 Anopheles aquasalis TSA
Fesavirus1 AII82258
Berkeley Virus (SRA dataset SRR070416; file S14)
GBEV01003876 Procambarus clarkii TSA
GAFS01012175 Pontastacus leptodactylus TSA
Halastavi arva RNA virus YP 004935381
Marine RNA virus JP-B YP 001429583
Halyomorpha halys virus YP 008719809
Cricket paralysis virus AAF80998
Drosophila C virus NP 044945
Solenopsis invicta virus-1 YP 164440
Acute bee paralysis virus AF486073
Formica exsecta virus 1 AHB62420
Israeli acute paralysis virus AEL12438
0.76
0.98
0.78
0.67
0.2

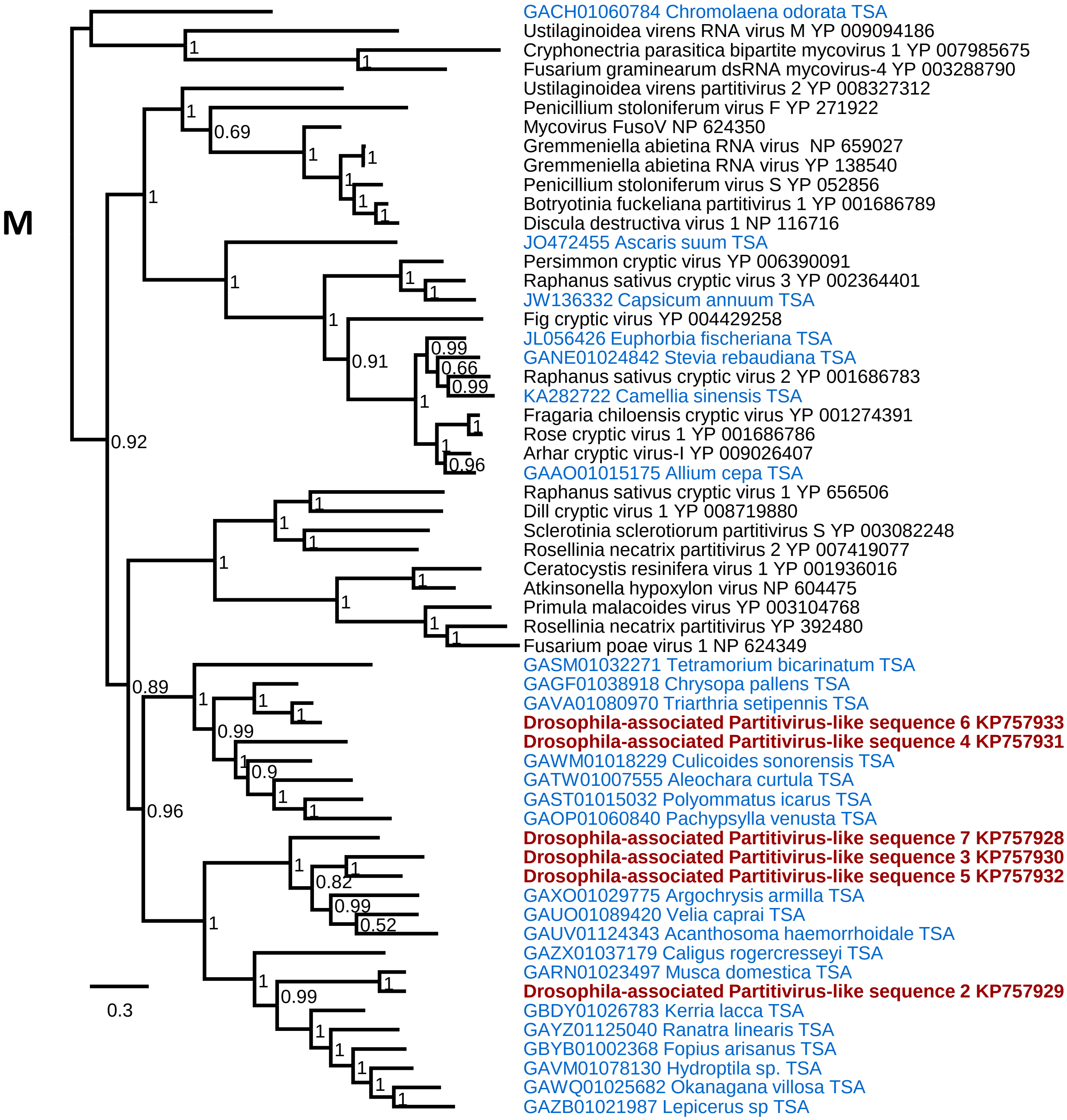
M
GACH01060784 Chromolaena odorata TSA
Ustilaginoidea virens RNA virus M YP 009094186
Cryphonectria parasitica bipartite mycovirus 1 YP 007985675
Fusarium graminearum dsRNA mycovirus-4 YP 003288790
Ustilaginoidea virens partitivirus 2 YP 008327312
Penicillium stoloniferum virus F YP 271922
Mycovirus FusoV NP 624350
Gremmeniella abietina RNA virus NP 659027
Gremmeniella abietina RNA virus YP 138540
Penicillium stoloniferum virus S YP 052856
Botryotinia fuckeliana partitivirus 1 YP 001686789
Discula destructiva virus 1 NP 116716
JO472455 Ascaris suum TSA
Persimmon cryptic virus YP 006390091
Raphanus sativus cryptic virus 3 YP 002364401
JW136332 Capsicum annuum TSA
Fig cryptic virus YP 004429258
JL056426 Euphorbia fischeriana TSA
GANE01024842 Stevia rebaudiana TSA
Raphanus sativus cryptic virus 2 YP 001686783
KA282722 Camellia sinensis TSA
Fragaria chiloensis cryptic virus YP 001274391
Rose cryptic virus 1 YP 001686786
Arhar cryptic virus-I YP 009026407
GAAO01015175 Allium cepa TSA
Raphanus sativus cryptic virus 1 YP 656506
Dill cryptic virus 1 YP 008719880
Sclerotinia sclerotiorum partitivirus S YP 003082248
Rosellinia necatrix partitivirus 2 YP 007419077
Ceratocystis resinifera virus 1 YP 001936016
Atkinsonella hypoxylon virus NP 604475
Primula malacoides virus YP 003104768
Rosellinia necatrix partitivirus YP 392480
Fusarium poae virus 1 NP 624349
GASM01032271 Tetramorium bicarinatum TSA
GAGF01038918 Chrysopa pallens TSA
GAVA01080970 Triarthria setipennis TSA
Drosophila-associated Partitivirus-like sequence 6 KP757933
Drosophila-associated Partitivirus-like sequence 4 KP757931
GAWM01018229 Culicoides sonorensis TSA
GATW01007555 Aleochara curtula TSA
GAST01015032 Polyommatus icarus TSA
GAOP01060840 Pachypsylla venusta TSA
Drosophila-associated Partitivirus-like sequence 7 KP757928
Drosophila-associated Partitivirus-like sequence 3 KP757930
Drosophila-associated Partitivirus-like sequence 5 KP757932
GAXO01029775 Argochrysis armilla TSA
GAUO01089420 Velia caprai TSA
GAUV01124343 Acanthosoma haemorrhoidale TSA
GAZX01037179 Caligus rogercresseyi TSA
GARN01023497 Musca domestica TSA
Drosophila-associated Partitivirus-like sequence 2 KP757929
GBDY01026783 Kerria lacca TSA
GAYZ01125040 Ranatra linearis TSA
GBYB01002368 Fopius arisanus TSA
GAVM01078130 Hydroptila sp. TSA
GAWQ01025682 Okanagana villosa TSA
GAZB01021987 Lepicerus sp TSA
0.3

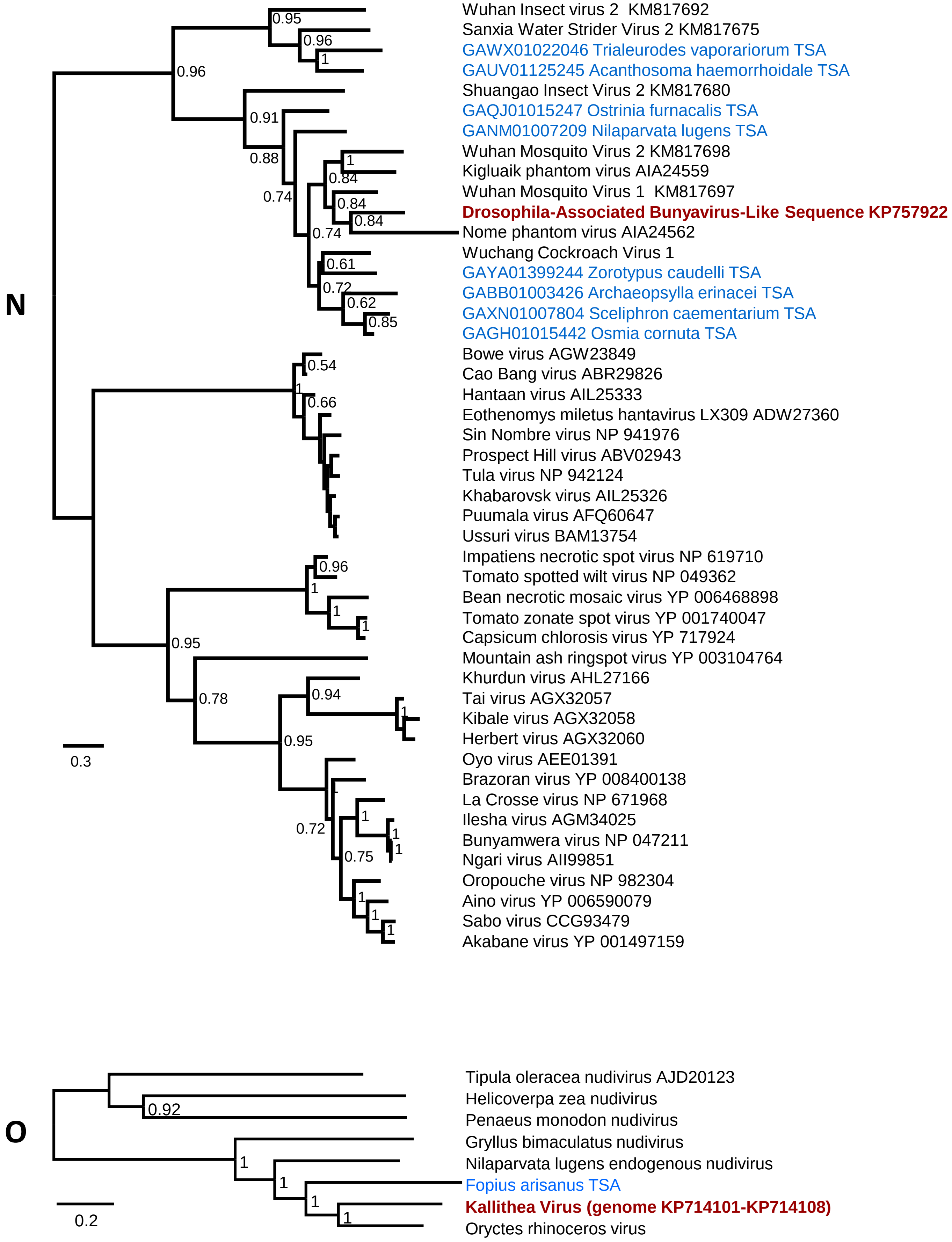
N
Wuhan Insect virus 2 KM817692
Sanxia Water Strider Virus 2 KM817675
GAWX01022046 Trialeurodes vaporariorum TSA
GAUV01125245 Acanthosoma haemorrhoidale TSA
Shuangao Insect Virus 2 KM817680
GAQJ01015247 Ostrinia furnacalis TSA
GANM01007209 Nilaparvata lugens TSA
Wuhan Mosquito Virus 2 KM817698
Kigluaik phantom virus AIA24559
Wuhan Mosquito Virus 1 KM817697
Drosophila-Associated Bunyavirus-Like Sequence KP757922
Nome phantom virus AIA24562
Wuchang Cockroach Virus 1
GAYA01399244 Zorotypus caudelli TSA
GABB01003426 Archaeopsylla erinacei TSA
GAXN01007804 Sceliphron caementarium TSA
GAGH01015442 Osmia cornuta TSA
Bowe virus AGW23849
Cao Bang virus ABR29826
Hantaan virus AIL25333
Eothenomys miletus hantavirus LX309 ADW27360
Sin Nombre virus NP 941976
Prospect Hill virus ABV02943
Tula virus NP 942124
Khabarovsk virus AIL25326
Puumala virus AFQ60647
Ussuri virus BAM13754
Impatiens necrotic spot virus NP 619710
Tomato spotted wilt virus NP 049362
Bean necrotic mosaic virus YP 006468898
Tomato zonate spot virus YP 001740047
Capsicum chlorosis virus YP 717924
Mountain ash ringspot virus YP 003104764
Khurdun virus AHL27166
Tai virus AGX32057
Kibale virus AGX32058
Herbert virus AGX32060
Oyo virus AEE01391
Brazoran virus YP 008400138
La Crosse virus NP 671968
Ilesha virus AGM34025
Bunyamwera virus NP 047211
Ngari virus AII99851
Oropouche virus NP 982304
Aino virus YP 006590079
Sabo virus CCG93479
Akabane virus YP 001497159
0.3
O
Tipula oleracea nudivirus AJD20123
Helicoverpa zea nudivirus
Penaeus monodon nudivirus
Gryllus bimaculatus nudivirus
Nilaparvata lugens endogenous nudivirus
Fopius arisanus TSA
Kallithea Virus (genome KP714101-KP714108)
Oryctes rhinoceros virus
0.2
