# Supplementary material for: The Discovery, Distribution, and Evolution of Viruses Associated with Drosophila melanogaster
Source: PLoS Biol. 2015 Jul 14;13(7):e1002210. doi: 10.1371/journal.pbio.1002210 (PMC4501690; doi:10.1371/journal.pbio.1002210)
Supplement: S1 Text — Output from the software package mirDeep [76] showing the proposed pre-miRNA hairpin, read numbers, and predicted folding pattern and energy. Reads are summed across all small-RNA libraries. (PDF) [file pbio.1002210.s028.pdf]

Provisional ID	:	Nudivirus_Part1_NonUK_37_541
Score total	:	5951.7
Score for star read(s)	:	3.9
Score for read counts	:	5947.7
Score for mfe	:	-1.4
Score for randfold	:	1.6
Score for cons. seed	:	
Total read count	:	11678
Mature read count	:	11581
Loop read count	:	1
Star read count	:	96

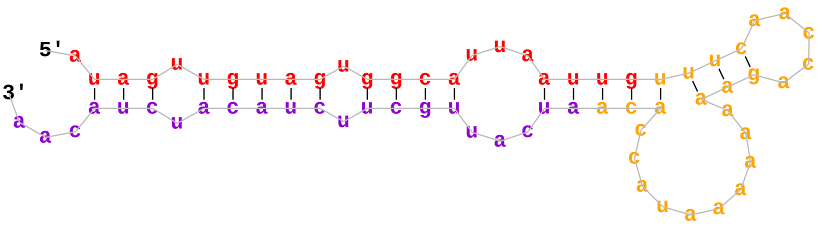

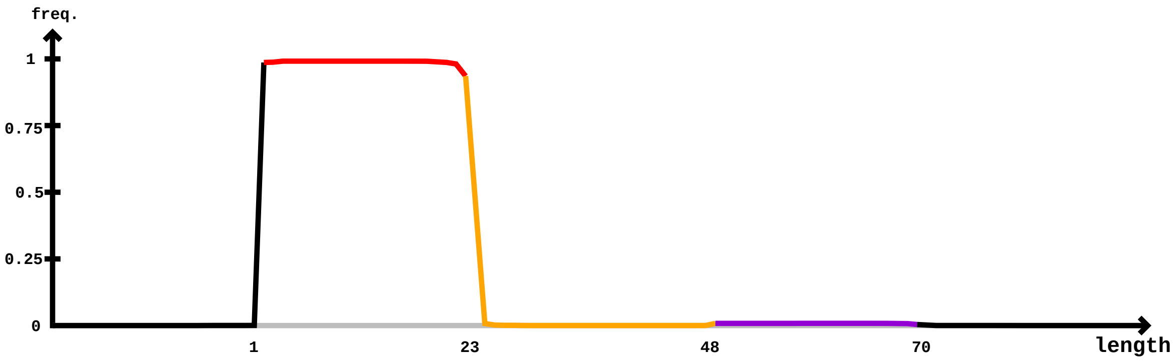

Mature Star

```
5'- uauuaguagcaucaauagugauaguuguaguggcauuaauuguuucaaccagaaaaaaaauaccacaaucauugcuucuacaucuacaacuacggcuaccauaauugcauca  -3'   obs
    uauuaguagcaucaauagugauaguuguaguggcauuaauuguuucaaccagaaaaaaaauaccacaaucauugcuucuacaucuacaacuacggcuaccauaauugcauca        exp
    .....(((((.....((((..(((.(((((.((((...((((((((.....)))..........)))))...)))).))))).)))..))))..))))).............   reads   mm   sample
    ..............auagugauaguuguagGggca.............................................................................   1       1    seq
    ..............auagugauaguuguaguggcC.............................................................................   1       1    seq
    ...............uagugauaguuguaguggcau............................................................................   1       0    seq
    ..................Agauaguuguaguggcauuaauu.......................................................................   1       1    seq
    ...................Cauaguuguaguggcauuaauug......................................................................   1       1    seq
    ...................Aauaguuguaguggcauuaauug......................................................................   1       1    seq
    ....................auaguuguaguggcauu...........................................................................   2       0    seq
    ....................auaguuguaguggcauuG..........................................................................   7       1    seq
    ....................auaguuguaguggcauua..........................................................................   11      0    seq
    ....................auaguuguaguggcauuU..........................................................................   7       1    seq
    ....................auaguuguaguggcauAa..........................................................................   1       1    seq
    ....................auaguuguaguCgcauuaa.........................................................................   1       1    seq
    ....................auaguuguaguggcauuaU.........................................................................   2       1    seq
    ....................auUguuguaguggcauuaa.........................................................................   1       1    seq
    ....................auaguuguaguggcauuaa.........................................................................   20      0    seq
    ....................auaguuguaguggcauuCau........................................................................   1       1    seq
    ....................auaguuguaguggcauuaau........................................................................   67      0    seq
    ....................auaAuuguaguggcauuaauu.......................................................................   1       1    seq
    ....................auaguuguaUuggcauuaauu.......................................................................   1       1    seq
    ....................auaguuguaguggcaCuaauu.......................................................................   1       1    seq
    ....................auagAuguaguggcauuaauu.......................................................................   1       1    seq
    ....................auaguuguaguUgcauuaauu.......................................................................   1       1    seq
    ....................auaguuguagugAcauuaauu.......................................................................   1       1    seq
    ....................auaguuguGguggcauuaauu.......................................................................   1       1    seq
    ....................Uuaguuguaguggcauuaauu.......................................................................   1       1    seq
    ....................auaguuguaguggcauuCauu.......................................................................   8       1    seq
    ....................auaguuguaguggcauuaauu.......................................................................   507     0    seq
    ....................auaguuguaguggcauuaauG.......................................................................   3       1    seq
    ....................auaguuguagCggcauuaauu.......................................................................   1       1    seq
    ....................auagGuguaguggcauuaauug......................................................................   3       1    seq
    ....................auaguuguaguggcauuaUuug......................................................................   3       1    seq
    ....................auagAuguaguggcauuaauug......................................................................   1       1    seq
    ....................auaguuguaguggcauuaauGg......................................................................   1       1    seq
```

```
                    Mature                                              Star
uauuaguagcaucaauagugauaguuguaguggcauuaauuguuucaaccagaaaaaaaaauaccacaaucauugcuucuacaucuacaacuacggcuaccauaaauugcauca
....................auaguuguagugAcauuaauug........................................................................ 2      1  seq
....................auaguuguagugCcauuaauug........................................................................ 2      1  seq
....................auaguuguagAggcauuaauug........................................................................ 1      1  seq
....................aCaguuguaguggcauuaauug........................................................................ 3      1  seq
....................auaguuguaguggcauuCauug........................................................................ 194    1  seq
....................auaguuguaguggcauAaauug........................................................................ 2      1  seq
....................auaguuCuaguggcauuaauug........................................................................ 2      1  seq
....................auaguuguaguggcauCaauug........................................................................ 2      1  seq
....................auaguuguaAuggcauuaauug........................................................................ 1      1  seq
....................auaguuguagGggcauuaauug........................................................................ 7      1  seq
....................aGaguuguaguggcauuaauug........................................................................ 2      1  seq
....................auaguuguaguggcauuGauug........................................................................ 4      1  seq
....................auaguuguaguUgcauuaauug........................................................................ 7      1  seq
....................auaguuguaguggcauuaGuug........................................................................ 2      1  seq
....................auaguuguagCggcauuaauug........................................................................ 7      1  seq
....................auaguuguaguggcauuaaAug........................................................................ 3      1  seq
....................auaguugCaguggcauuaauug........................................................................ 2      1  seq
....................auaguAguaguggcauuaauug........................................................................ 1      1  seq
....................auaguuguaguggGauuaauug........................................................................ 1      1  seq
....................auaguuguaguggcauuaauug........................................................................ 10040  0  seq
....................auaguuguaCuggcauuaauug........................................................................ 8      1  seq
....................auaguuguaguggcauuaauuC........................................................................ 4      1  seq
....................auaguuguaguCgcauuaauug........................................................................ 5      1  seq
....................auGguuguaguggcauuaauug........................................................................ 1      1  seq
....................auaguuguaguggcauuaaCug........................................................................ 1      1  seq
....................auagCuguaguggcauuaauug........................................................................ 2      1  seq
....................Uuaguuguaguggcauuaauug........................................................................ 1      1  seq
....................auaguuguaUuggcauuaauug........................................................................ 10     1  seq
....................auaguuAuaguggcauuaauug........................................................................ 3      1  seq
....................auaguuguaguggcCuuaauug........................................................................ 3      1  seq
....................auaguuguaguggcauuUauug........................................................................ 1      1  seq
....................auaguuguUguggcauuaauug........................................................................ 1      1  seq
....................auaguuguaguggUauuaauug........................................................................ 1      1  seq
....................auaguuUuaguggcauuaauug........................................................................ 7      1  seq
....................auaguuguaguggAauuaauug........................................................................ 1      1  seq
....................auaCuuguaguggcauuaauug........................................................................ 6      1  seq
....................auaguuguaguggcUuuaauug........................................................................ 3      1  seq
....................auaguuguaguggcauuaauuA........................................................................ 6      1  seq
....................auCguuguaguggcauuaauug........................................................................ 2      1  seq
....................auaguugGaguggcauuaauug........................................................................ 1      1  seq
....................auaguuguagugUcauuaauug........................................................................ 4      1  seq
....................Guaguuguaguggcauuaauug........................................................................ 3      1  seq
....................auaguuguaguAgcauuaauug........................................................................ 3      1  seq
....................auaguuguaguggcauuaauuU........................................................................ 43     1  seq
....................auaguuguaguggcauuaCuug........................................................................ 3      1  seq
....................auaUuuguaguggcauuaauug........................................................................ 13     1  seq
....................auaguuUuaguggcauuaauugu....................................................................... 1      1  seq
....................auaguuguaguggcauuaauuUu....................................................................... 3      1  seq
....................auaguuguaguggcauuCauugu....................................................................... 12     1  seq
....................auaguuguaguggcauuaauugC....................................................................... 2      1  seq
....................auaguuguaguggcauuaauugu....................................................................... 323    0  seq
....................auaguuguaguggcauuaauugA....................................................................... 20     1  seq
....................auaguuguaguggcauuaauuguG...................................................................... 1      1  seq
....................auaguuguaguggcauuaauuUuu...................................................................... 3      1  seq
....................auaguuguaguggcauuaauuguA...................................................................... 1      1  seq
....................auaguuguaguggcauuCauuguu...................................................................... 1      1  seq
....................auaguuguaguggcauuaauuguu...................................................................... 54     0  seq
....................Guaguuguaguggcauuaauuguu...................................................................... 1      1  seq
....................auaguuguaguggcauuaauuguuu..................................................................... 12     0  seq
....................auaguuguaguggcauuaauuguuA..................................................................... 1      1  seq
....................auaguuguaguggcauuaauuguuuc.................................................................... 1      0  seq
....................auaguuguaguggcauuaauuguuuca................................................................... 7      0  seq
....................auaguuguaguggcauuaauuguuucaa.................................................................. 1      0  seq
....................auaguuguaguggcauuCauuguuucaa.................................................................. 1      1  seq
.....................uaguuguaguggcauuaauu......................................................................... 2      0  seq
.....................uaguuguaguggcauuaauug........................................................................ 8      0  seq
.....................uaguuguaguggcauuaauugu....................................................................... 2      0  seq
.....................uaguuguaguggcauuaCuuguu...................................................................... 1      1  seq
.....................uaguuguaguggcauuaauuguu...................................................................... 1      0  seq
.....................uaguuguaguggcauuaauuguA...................................................................... 1      1  seq
```

Mature **Star**

```
uauuaguagcaucaauagugauaguuguaguggcauuaauuguuucaaccagaaaaaaaauaccacaaucauugcuucuacaucuacaacuacggcuaccauaauugcauca
......................aUuuguaguggcauuaauu.......................................................................   3   1   seq
......................aguuguaguggcauuaauug......................................................................   2   0   seq
......................aUuuguaguggcauuaauug......................................................................   37  1   seq
......................aguuguaguggcauuaauugu.....................................................................   1   0   seq
......................aguuguaguggcauuaauuguu....................................................................   1   0   seq
..........................................uuucaaccagaaaaaaaauaccaca.............................................   1   0   seq
..................................................................aaucauugcuucuacaucuaca........................   2   0   seq
...................................................................aucauugcuucuacauc............................   1   0   seq
...................................................................aucauugcuucuacaucu...........................   1   0   seq
...................................................................aucauugcuucuacaucua..........................   6   0   seq
...................................................................aucauugcuucuacaucuaU.........................   3   1   seq
...................................................................aucauugcuucuacaucuac.........................   2   0   seq
...................................................................aucauugcuucuacaucuacC........................   3   1   seq
...................................................................aucauugcuucuacaucuaUa........................   2   1   seq
...................................................................aucauugcuucuacaucuaca........................   31  0   seq
...................................................................aucCuugcuucuacaucuaca........................   1   1   seq
...................................................................aucauugcuucuacaucuacaa.......................   33  0   seq
...................................................................aucauugcuucuacaucuacaC.......................   1   1   seq
...................................................................aucauugcuucuacaucuaUaa.......................   8   1   seq
...................................................................aucauugcuucuacaucuacCa.......................   2   1   seq
............................................................................ucuacaucuacaacuacggcu...............   1   0   seq
............................................................................ucuacaucuacaacuacggcuaA.............   1   1   seq
```
