# Supplementary material for: The Discovery, Distribution, and Evolution of Viruses Associated with Drosophila melanogaster
Source: PLoS Biol. 2015 Jul 14;13(7):e1002210. doi: 10.1371/journal.pbio.1002210 (PMC4501690; doi:10.1371/journal.pbio.1002210)
Supplement: S2 Text — (DOCX) [file pbio.1002210.s029.docx]

**Standard Protocol for PCR assays**

**PCR cycle:**

| Temp | Time | Description |
| --- | --- | --- |
| 94C | 5 min | initial denaturing |
|  |  |  |
| Cycles of |  |  |
| 94C | 15s | denaturing |
| see assay | 30s | annealing |
| 72C | see assay | extension |
|  |  |  |
| 72C | 5 min | final extension |

**For a single reaction**

| Ingredient | Concentration | Volume |
| --- | --- | --- |
|  |  |  |
| 10x NH_4_-based Reaction Buffer | 10x | 2 µl |
| dNTPs | 10mM total | 0.6 µl |
| MgCl2 | 50mM | 0.5 µl |
| primer F | 10 uM | 1 µl |
| primer R | 10 uM | 1 µl |
| taq | 5U/µl | 0.1 µl |
| template |  | 2 µl |
| triple distilled (3’D) H20 | | 15 µl |

For multiplex assays reduce water, use equal quantity of primers

**Bloomfield Virus (Related to reoviridae)**

**Assay 1**

Reo1_S7_qPCR_2F_494 AATAAATCATAAATTTTTGGACTCAGATTGG

Reo1_S7_qPCR_4R_1397 AATAAATCATAAGCCAAAATACTTGTTCCAG

**Product ~ 900bp**

Anneal at 60 degrees with 1m extension for 35 cycles.

**Assay 2**

Reo1_S4_1F_620 AATAAATCATAACTATGGTTATCGATTGCATGGTCC

Reo1_S4_2R_1636 AATAAATCATAAGTAAACAAATCAAAACCATC

**Product ~ 1000bp**

Anneal at 53 degrees with 1m extension for 35 cycles.

*Assay 1 and Assay 2 target different virus segments. The 5' bases marked in grey do not target the template, and are intended to increase sensitivity and specificity when primers are used for qPCR.*

**Chaq Virus**

222_140F AACAGAACGWCTGCTTTTTGGAAATCC

222_660R TCCATGTCCTGTHGGGTCTATCTG

**Product ~ 520bp**

Anneal at 55 degrees with 45s extension for 35 cycles.

**Craigie’s Hill Virus (Related to Nodaviruses)**

**Assay 1**

NV-L_1.69F GCAAAATCCGTGGTTCATACCAG

NV-L_2.89R CTTAACAGGACGCTCCAAGTGGAT

**Product ~ 1200bp**

Anneal at 55 degrees with 1m 30s extension for 35 cycles.

**Assay 2**

674_100F CCTATCTGTCAAGCTGTWCTGCCAAC

674_1540R GTGTGCCAACTAGGCTCAGGAG

**Product ~ 1440bp**

Anneal at 57 degrees with 1m 30s extension for 35 cycles.

**Dansoman Virus (Related to CBPV)**

**Assay 1**

CBP1-L_0.90F GCGCAGACGGAGGACGGCA

CBP1-L_2.27R ARCGGKGTCACWCGCGGCTC

**Product ~ 1300bp**

Anneal at 61 degrees with 1min extension for 35 cycles.

*Assay 1 is the preferred assay for Dansoman virus, but it doesn’t detect all haplotypes, and has given false-positive bands. Since the virus is bipartite, look for agreement between all assays for each sample.*

**Assay 2**

CBP1-1.1F GGCTCAACAACAACmGGTACGCGAT

CBP1-1.8R CATGCTGCCTATCAGyCTGGGGC

**Product ~ 700bp**

Anneal at 57 degrees with 2min extension for 35 cycles.

**Assay 3**

CBP1_con_1740F GACRTRTCCAACACYAGRCCCGACA

CBP1_con_3720R TGCCyTTAATCArAAACGGGCA

**Product ~ 900bp**

Anneal at 57 degrees with 2min extension for 38 cycles.

**Assay 4**

CBP2_new_1090F ACTCGCTAGCTGCYGCGAGTTG

CBP2_new_1530R GTGGTATTGCAGTCAGGYACAGTCC

**Product ~ 500bp**

Run at 59 degrees with 1min extension for 35 cycles.

**Assay 5**

CBP2_new_1190F TTGGCACGGGAATGACTGCCTT

CBP2_new_2000R CCTGACACCTTGTGGCATGTTGG

**Product ~ 900bp**

Anneal at 59 degrees with 1min extension for 35 cycles.

**Drosophila A Virus (Related to Permutotetraviridae)**

This is a multiplex assay

DAV8F AGAGAGAATCTTGATCCCTCCCT

DAV 540R AAGCCATCCCAAGTGCCTCCTGT

**Product ~ 530bp**

DAV 3300F TGCAAGTAAGCTCTTGCCAACCCT

DAV 3940R AGATACCACTTACGGGTGGTTGC

**Product ~ 640bp**

Anneal at 55 degrees with 1 min extension for 35 cycles.

**Drosophila C Virus (Dicistroviruses)**

**Assay 1**

DCV7 AGTATGATTTTGATGCAGTTGAATCTC

DCV8 GAAGCACGATACTTCTTCCAAACC

**Product ~ 524bp**

See Kapun et al., for cycling conditions (Kapun, Nolte, Flatt, & Schlötterer, 2010)

**Assay 2**

DCV1290F GATGGTGTTGGCTCTGAACAGATG

DCV1590R CAACTGTATCTTCCAATGCACCCTG

**Product ~ 300bp**

See Longdon et al., for cycling conditions (Longdon, Fabian, Hurst, & Jiggins, 2012)

**Galbut Virus**

**Assay 1**

407_170F GATCGAGATGGAACTCCRCTCTC

407_750R GCCKCATACTTGGTGCTGCCAACTG

**Product ~ 580bp**

Anneal at 55 degrees with 1m extension for 35 cycles.

**Assay 2**

543_160F GACAGAGCTGGTCHGTCTTGGG

543_770R CGGTATTCCAAACGTGCCTGATGG

**Product ~ 610bp**

Run at 55 degrees with 1m extension for 35 cycles.

**Kallithea Virus (DNA Nudivirus)**

This is a multiplex assay. Both fragments target expressed mRNAs, and can be run on DNA or cDNA

NudiPif1_F CGACATCACATTCGACCCATATCC

NudiPif1_R TCCCATAAAGTGCGATCCCATAG

**Product ~ 970bp**

NudiPol_F CCACGGTAATCGAGAGTATTTCG

NudiPol_R GTCAGGTACGACATCATATCCATC

**Product ~ 1050bp**

Run at 57 degrees with 1m 30s extension for 35 cycles.

Examine on a 2% gel to resolve double banded product.

**La jolla Virus (Related to Iflaviruses)**

SB-L_6.77_F GTGGAGTAAAGCAACGACTTGG

SB_ASSAY_1R CAACTGCRTGTTTGAGTTCCCAACGA

**Product ~ 1300bp**

Anneal at 55 degrees with 1m 30s extension for 35 cycles.

**Motts Mill Virus**

**Assay 1**

105_160F AATCGCTCCAMYCCAGGCACTAC

105_1080R TGGTAGCTGTYTTCTGRGCAGC

**Product ~ 920bp**

Anneal at 55 degrees with 1 min extension for 35 cycles.

**Assay 2**

221_100F ACAGCAGABTTCTTGCGAGSAGC

221_795R GACTGCCAYGTCTCATGCTTCACTTC

**Product ~ 695bp**

Anneal at 59 degrees with 45s extension for 35 cycles.

**Assay 3**

Luteo_1F_364 AATAAATCATAAATCGTGCTTGTTTCCTTGGC

Luteo_2R_1758 AATAAATCATAAGGTTGAACCAGTCGGTGAAT

**Product ~ 1400bp**

Anneal at 62 degrees with 1m extension for 35 cycles.

*Assays2 and 3 overlap. The 5' bases marked in grey for assay 3 do not target the template, and are intended to increase sensitivity and specificity when primersare used for qPCR.*

**Newfield Virus (Related to Permutotetraviridae)**

EEV_1F_1138 AATAAATCATAAGGCTGTTACGGTGATGATGG

EEV_4R_2213 AATAAATCATAACCGCTGAAAATACCGCTCA

**Product ~ 1075bp**

Anneal at 63 degrees with 1m extension for 35 cycles.

*The 5' bases marked in grey do not target the template, and are intended to increase sensitivity and specificity when primersare used for qPCR.*

**Nora Virus**

Nora 6220F GACCATTGGCACAAATCACCATTTG

Nora 7210R TCTTAGGCCGGTTGTCTTCACCC

**Product ~ 990bp**

Anneal at 55 degrees with 1 min extension for 35 cycles.

**DMelSV (Rhabdoviridae)**

Sigma5020F GGATTCAAAACCCTTTAATATCTGGCCT

Sigma5620F CCTGACATCAAGACGTAAACCTCTGA

**Product ~ 600bp**

Anneal at 55 degrees with 1m extension for 35 cycles.

**Thika Virus (Related to Picornavirales)**

Cripa_C3_5625F CTTCGAAGCATCYCTGCATCGTAAAG

Cripa_univ_6560R GCACCCACAGCTAGCATRTCTGG

**Product ~ 900bp**

Anneal at 55 degrees with 1 min extension for 35 cycles.

**torrey pines Virus (Related to reoviridae)**

345_425F GACGTCVTACATCAACGCTAACACGG

345_905R CACGACTGCAGGAGCATCATTAAC

**Product ~ 480bp**

Anneal at 61 degrees with 1m extension for 35 cycles.

**Twyford Virus (related to Iflaviruses)**

This assay is a multiplex PCR.

SB-L_1.51_F CGCAGTCAGTTTGCATCAGG

SB.TWY_2.57_R CTCAGCTAAGGAGCCTTCCAT

**Product ~ 900bp**

SB-L_5.22_F AGGGCTTTCCGGTGGGATG

SB.TWY_6.20_R AGCGGACAGGTTTCATACTGACA

**Product ~ 980bp**

Anneal at 57 degrees with 1m 30s extension for 35 cycles.

2% gel required to separate bands.

**Standard Protocol for qRT PCR assays**

**PCR cycle:**

| Temp | Time | Description |
| --- | --- | --- |
| 95C | 5 min | initial denaturing |
|  |  |  |
| 40 Cycles of |  |  |
| 95C | 5s | denaturing |
| 60C | 30s | Annealing/extension |

**For a single reaction**

| Ingredient | Concentration | Volume |
| --- | --- | --- |
|  |  |  |
| SensiFAST SYBR and Flourescein Mix | 10x | 7.5 µl |
| primer F | 10 uM | 1 µl |
| primer R | 10 uM | 1 µl |
| template |  | 2.5 µl |
| triple distilled (3’D) H20 | | 3 µl |

**qRT PCR assays**

**Drosophila melanogaster rPL32**

RpL32_qPCR_F_flap AATAAATCATAATGCTAAGCTGTCGCACAAATGG

RpL32_qPCR_R_flap AATAAATCATAATGCGCTTGTTCGATCCGTAAC

**Product ~ 110b**

*89% efficiency calculated from mean of standard curves*

**Drosophila C Virus (Dicistroviridae)**

DCV_qPCR_2F_flap AATAAATCATAAGCCACTGTGATTGATACAACAGAC

DCV_qPCR_2R_flap AATAAATCATAAGAAGCACGATACTTCTTCCAAACC

**Product ~ 115bp**

*96% efficiency calculated from mean of standard curves*

**Drosophila A Virus (Related to Permutotetraviridae)**

DAV_qPCR_2F_flap AATAAATCATAACCATCGTCACAACCACAAAGC

DAV_qPCR_2R_flap AATAAATCATAAGACGAGAAGATACGCTCTGTACG

**Product ~ 104bp**

*100% efficiency calculated from mean of standard curves*

**Nora Virus**

Nora_qPCR_3F_flap AATAAATCATAAGGTGTAGCAGGTCGTATTCTGC

Nora_qPCR_3R_flap AATAAATCATAACAATGGCTGAAACTGCTGTTCCTGC

**Product ~ 120bp**

*88% efficiency calculated from mean of standard curves*

**DMelSV (Rhabdoviridae)**

DMelSV_F_flap AATAAATCATAATTCAATTTTGTACGCGGAATC

DMelSV_R_flap AATAAATCATAATGATCAAACCGCTAGCTTCA

**Product ~ 139bp**

*83% efficiency calculated from mean of standard curves*

**Thika Virus (Related to Picornavirales)**

Cripa_C3_qPCR_1F_flap AATAAATCATAAGAGCTCAAAATGGATCTTGCC

Cripa_C3_qPCR_1R_flap AATAAATCATAAGGGTTGTGGATTCGTTTGTGATG

**Product ~ 176bp**

*93% efficiency calculated from mean of standard curves*

**Motts Mill Virus**

Luteo_qPCR_1F_364 AATAAATCATAAATCGTGCTTGTTTCCTTGGC

Luteo_qPCR_1R_489 AATAAATCATAAAGCCATCGAGGGTATGAGTC

**Product ~ 125bp**

*95% efficiency calculated from mean of standard curves*

**torrey pines Virus (Related to reoviridae)**

345_qPCR_1F_flap AATAAATCATAATACATCAACGCTAACACGG

345_qPCR_1R_flap AATAAATCATAATGGACGCATCAGTGAATAT

**Product ~ 158bp**

*92% efficiency calculated from mean of standard curves*

**Newfield Virus (Related to tetraviridae)**

EEV_qPCR_5F_2707 AATAAATCATAAATGGTTGAAGCCGCCAATC

EEV_qPCR_5F_2807 AATAAATCATAAACACCACCTTTCACCTTTGCGT

**Product ~ 100bp**

*92% efficiency calculated from mean of standard curves*

**Twyford Virus (related to Iflaviruses)**

Ifla_Twy_qPCR_1F_flap AATAAATCATAAATGGAGTTTCCCTTACACCAAC

Ifla_Twy_qPCR_1R_flap AATAAATCATAAGGCATGTGAGTTATCCAATGTG

**Product ~ 131bp**

*107% efficiency calculated from mean of standard curves*

**La jolla Virus (Related to Iflaviruses)**

IflaB_qPCR_4F_928 AATAAATCATAAGCTCTGATATCCCGGCC

IflaB_qPCR_4R_1064 AATAAATCATAAGCAGCTTTTGAACCATATTGTG

**Product ~ 136bp**

*92% efficiency calculated from mean of standard curves*

**Craigie’s Hill Virus (Related to Nodaviruses)**

noda_qPCR_1F_flap AATAAATCATAACGTTATGATTTATTCGTGGGCG

noda_qPCR_1R_flap AATAAATCATAAGCGTCAAATATAATAGGTGCC

**Product ~ 133bp**

*75% efficiency calculated from mean of standard curves*

**Melt curves for qPCR assays**

| 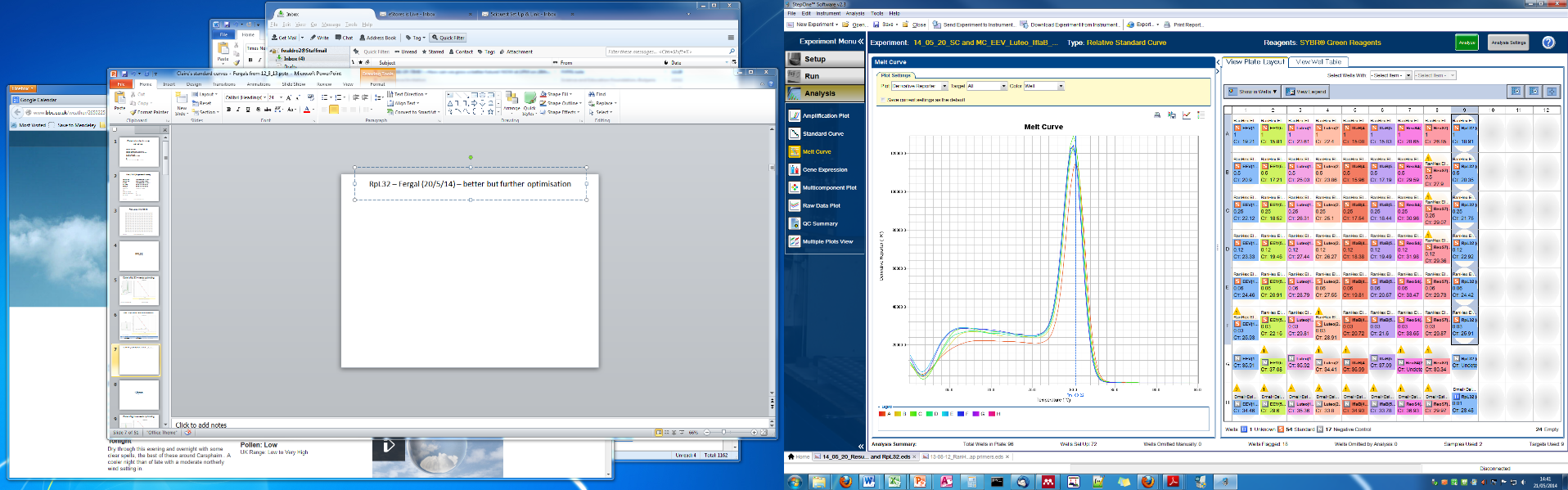  RpL32 | 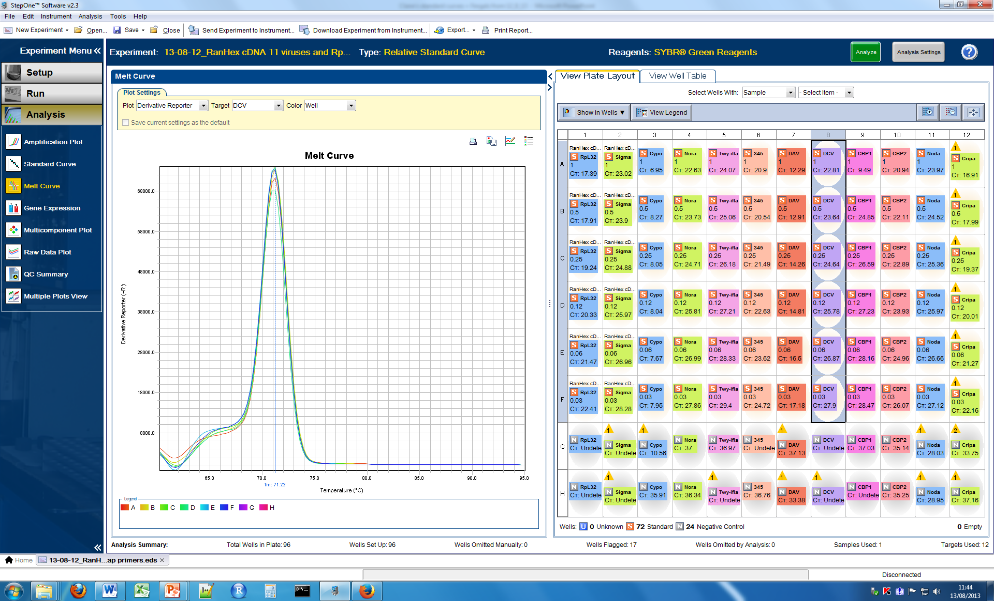  Drosophila C Virus | 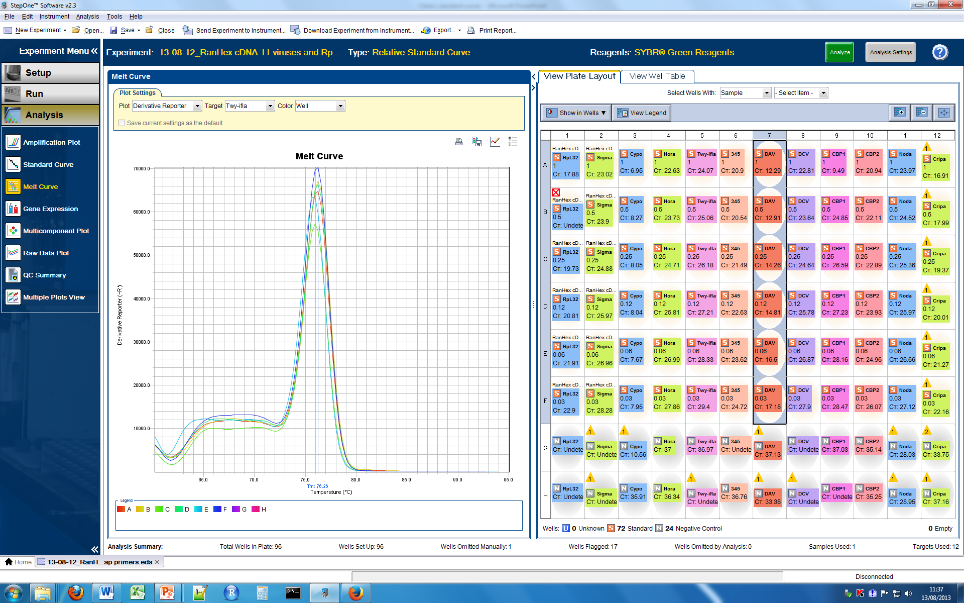  Drosophila A Virus |
| --- | --- | --- |
| 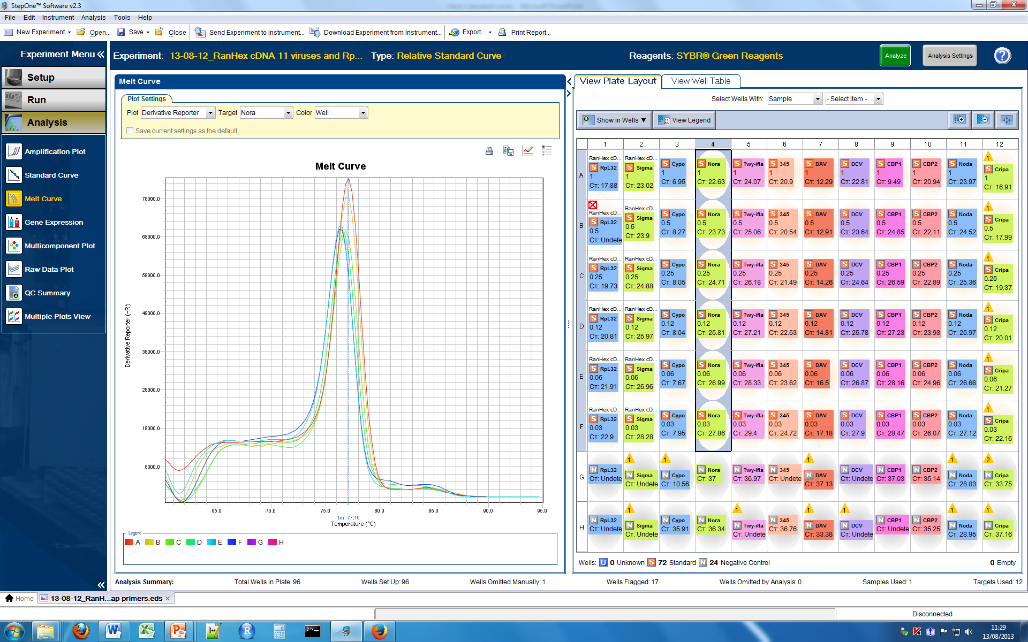  Nora Virus | 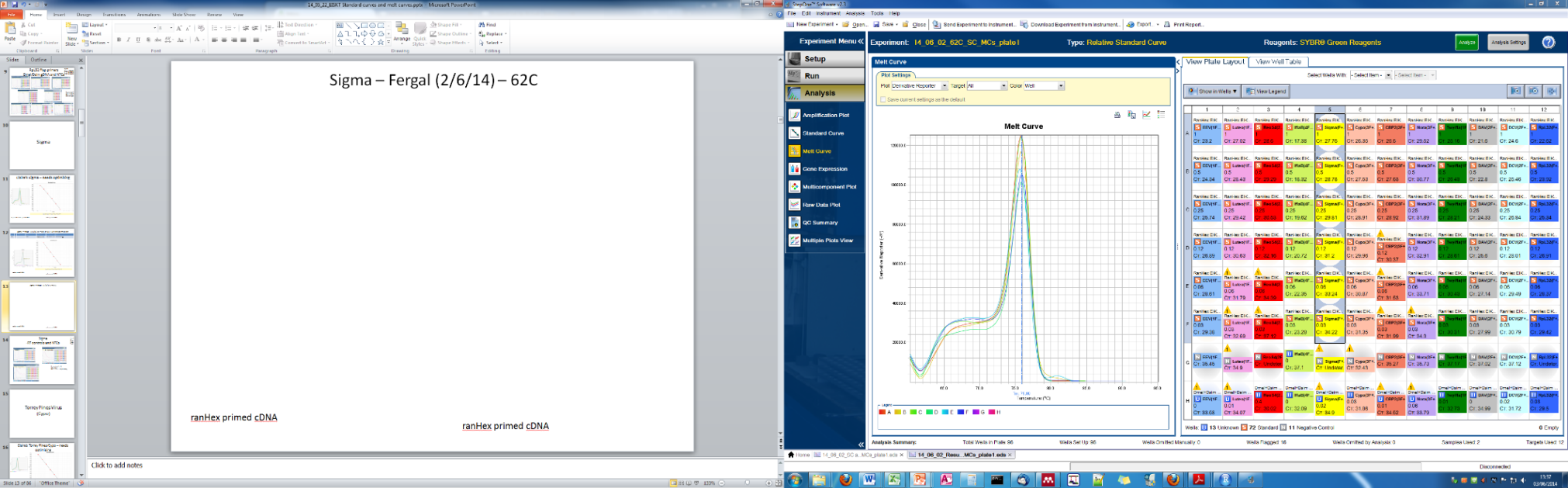DMelSV Virus | 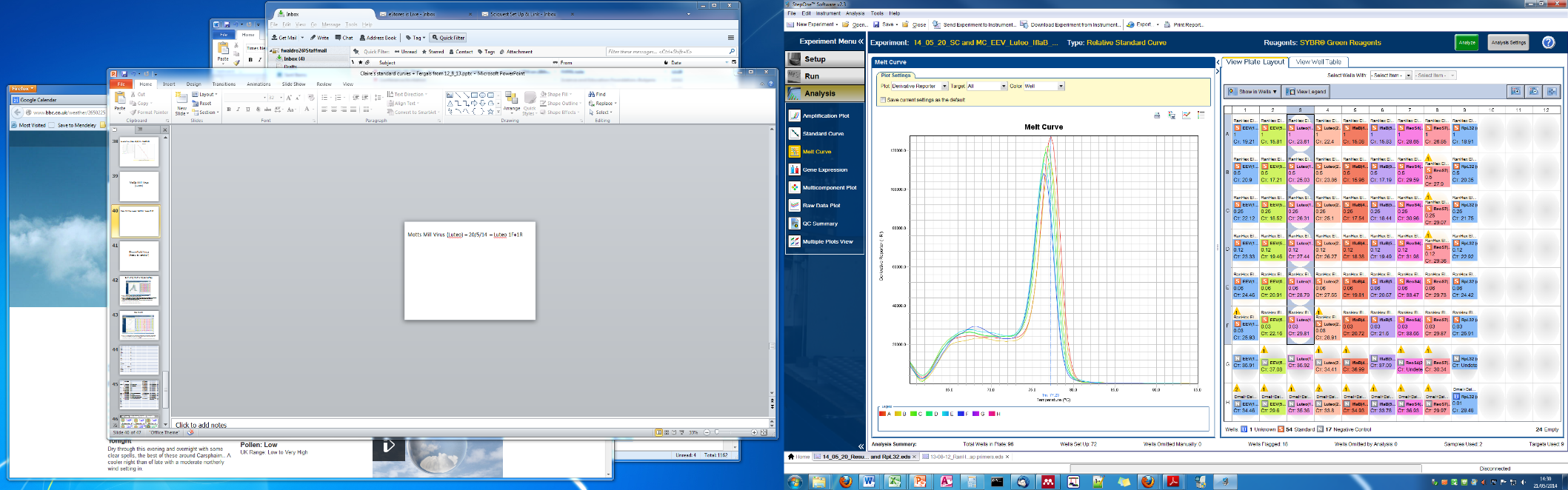  Motts Mill Virus |
| 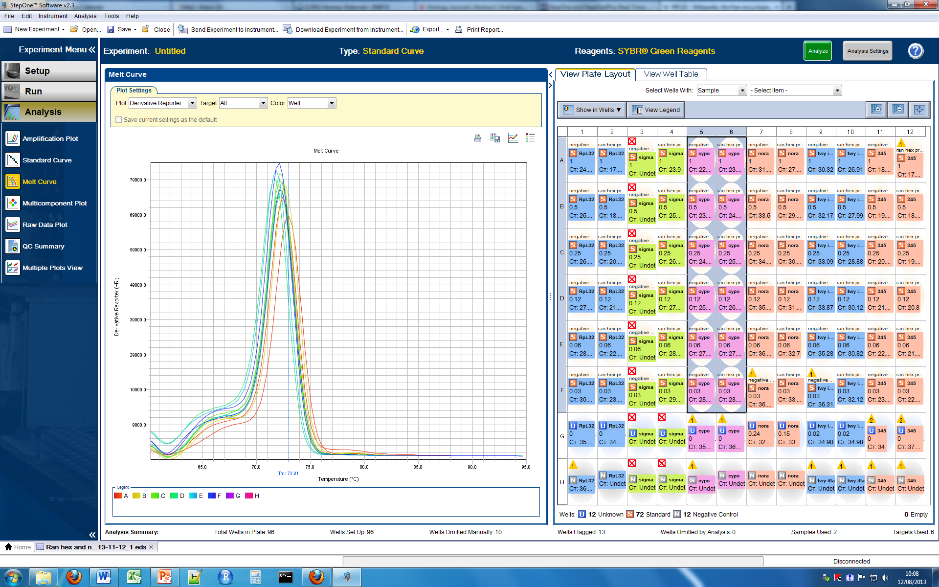  Torrey Pines Virus | 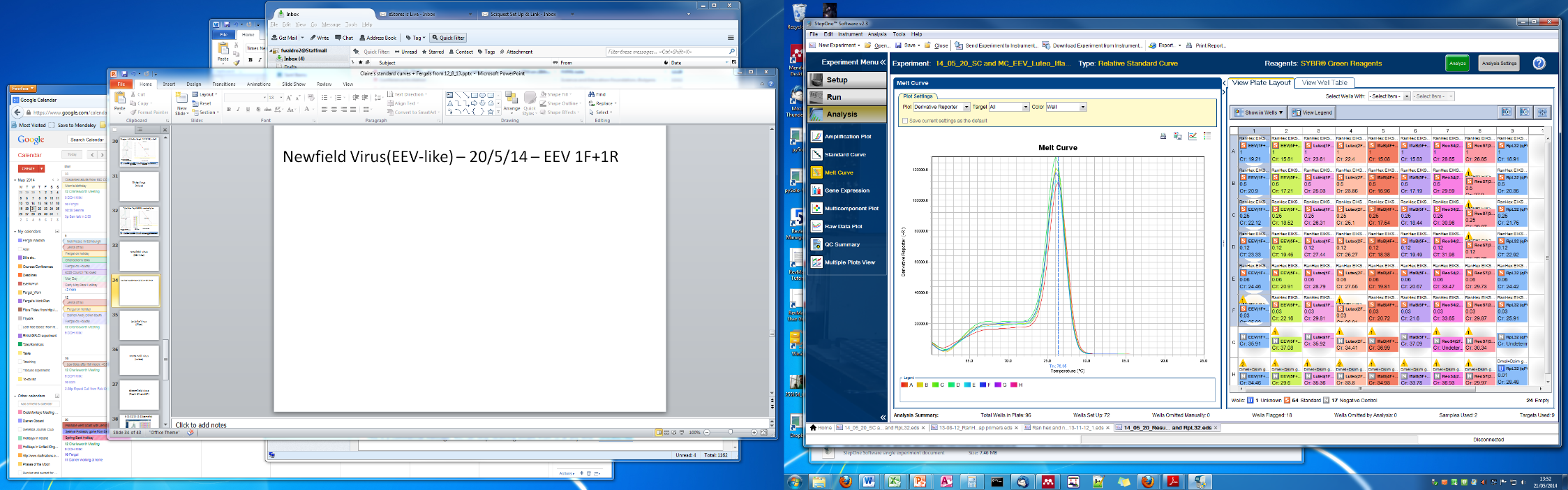  Newfield Virus | 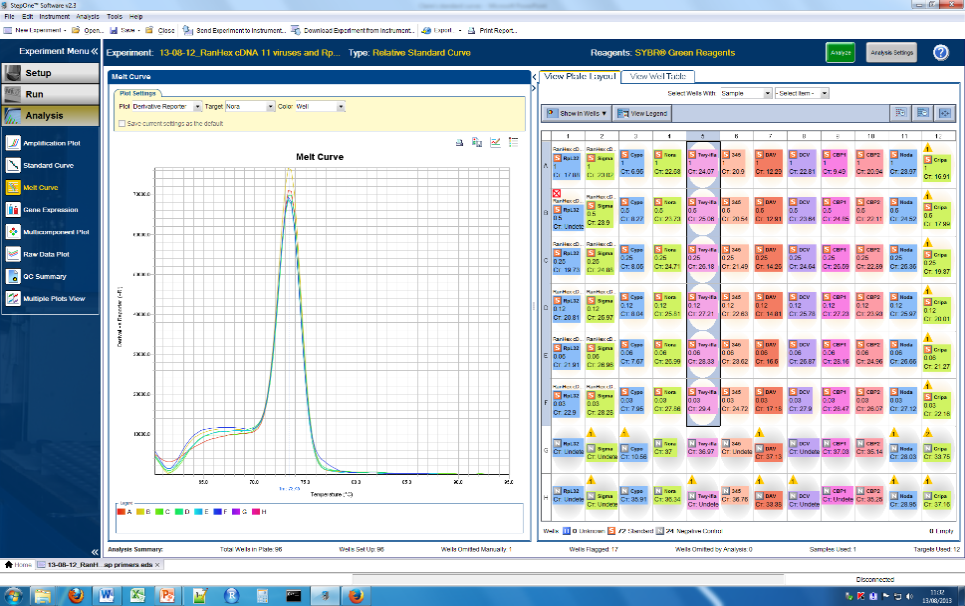  Twyford Virus |
| 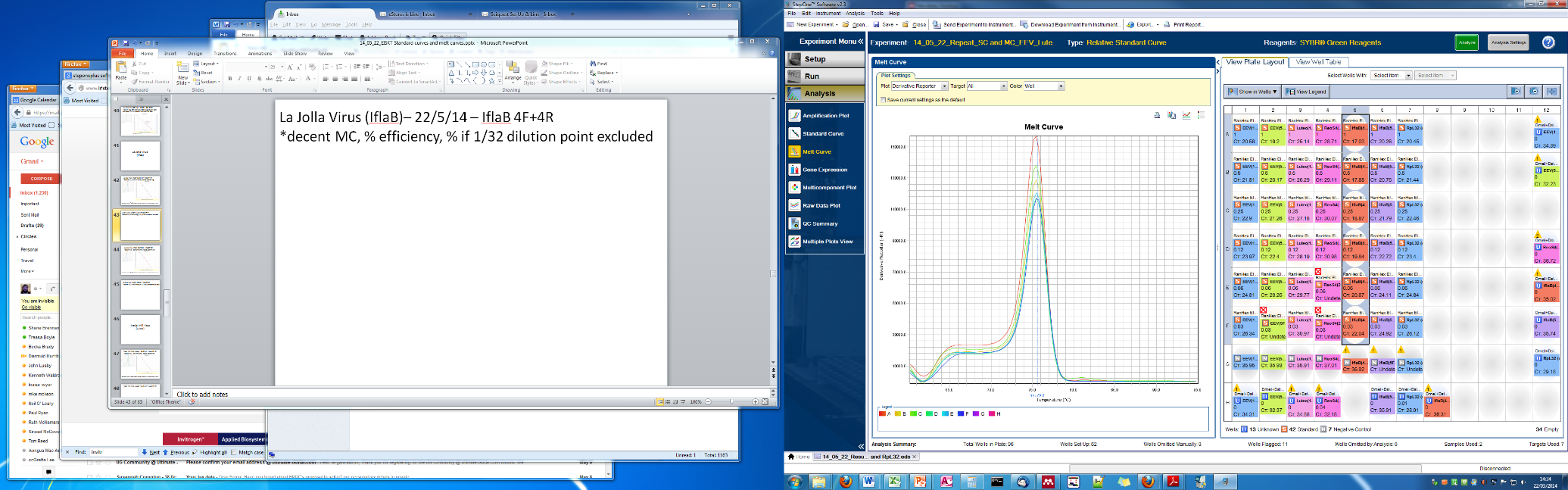  La Jolla Virus | 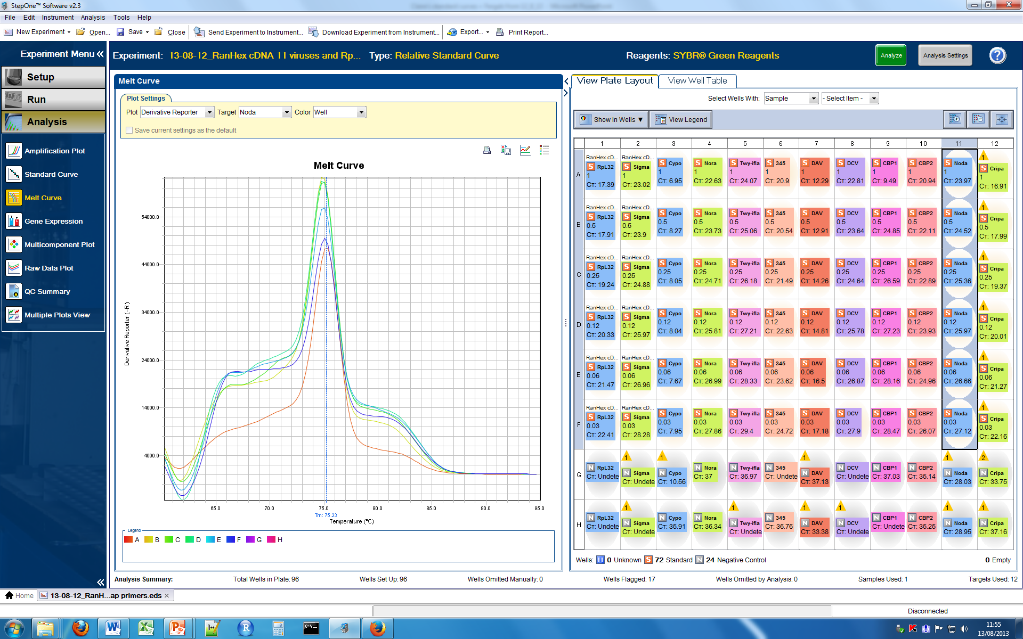  Craigie’s Hill Virus |  |

*Derivative reporter fluorescence is plotted against PCR amplicon melting temperature along a two-fold dilution series (rainbow red to blue)*
